# CD-MOF-1 for CO_2_ Uptake: Remote and Hybrid Green Chemistry Synthesis of a Framework Material with Environmentally Conscious Applications

**DOI:** 10.1021/acs.jchemed.2c00922

**Published:** 2023-02-13

**Authors:** Georganna Benedetto, Brittany M. Cleary, Colin T. Morrell, Claudia G. Durbin, Anna L. Brinks, John Tietjen, Katherine A. Mirica

**Affiliations:** † Department of Chemistry, Dartmouth College, Burke Laboratory, 41 College St., Hanover, New Hampshire 03755, United States; ‡ Lebanon High School, 195 Hanover St., Lebanon, New Hampshire 03766, United States

**Keywords:** Undergraduate and High School Experiment, Metal−Organic Framework, Environmental Application, Green Chemistry

## Abstract

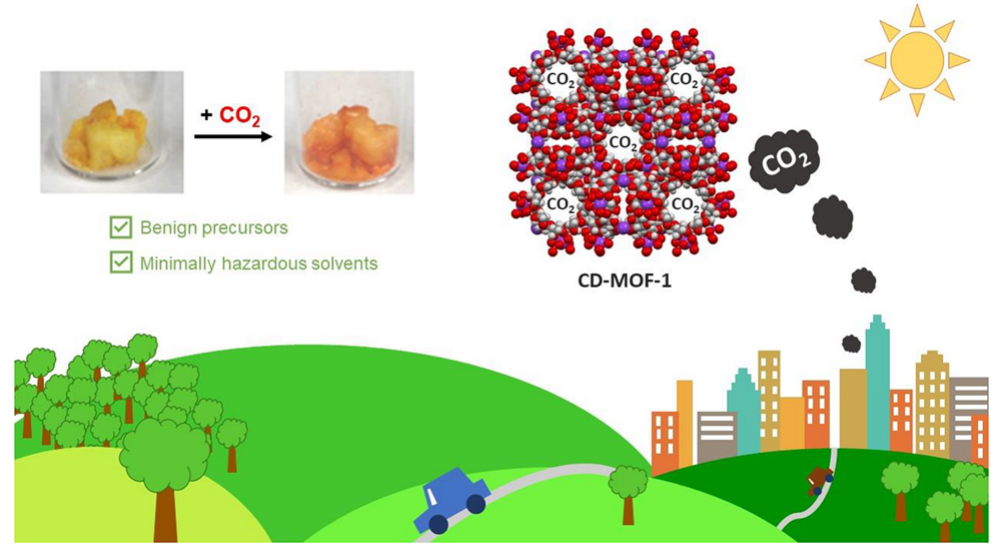

The chemistry of metal–organic frameworks (MOFs) has the potential to introduce high school and undergraduate students to the fundamental chemical principles of structure and bonding, enhance the development of skills in synthesis and crystal growth, and promote hands-on experience with gas capture and host–guest chemistry of emerging materials with desirable environmental applications. However, most available experiments in the pedagogical literature involving MOFs require laboratory equipment and the use of hazardous chemicals to facilitate crystal growth and the study of structure–property relationships. To remedy this gap in the literature, this paper describes an adapted experimental approach designed specifically for a household environment or low-resource laboratory to grow, activate, and use cyclodextrin-based MOFs for CO_2_ uptake. This experiment implements a simple procedure that can be carried out safely without access to specialized equipment or laboratory infrastructure. Despite the simplicity of the experimental design, this experiment presents an intellectually engaging opportunity for high school and undergraduate students to explore crystal growth and nucleation, coordination chemistry, and host–guest chemistry as well as green chemistry concepts such as the choice of benign reagents and solvents, and applications of porous materials for gas uptake.

The increased release of anthropogenic greenhouse gases (GHGs) such as CO_2_ has been directly linked to climate change as the GHGs form an effective insulating layer in the planet’s atmosphere.^1,2^ These GHGs produce a positive radiative force, meaning that the solar energy entering the atmosphere is larger than the energy that radiates into space, and results in climate change.^3^ Climate change poses a threat to the environment and society by increasing extreme weather patterns, resulting in food insecurity, loss of biodiversity, and mass extinction.^4,5^ As society and industries rely heavily on the combustion of fossil fuels, creating solutions to mitigate the CO_2_ released into the atmosphere is necessary.

One approach to mitigate CO_2_ emissions is the sequestration of the gas by either chemical or physical adsorbent materials. Chemical adsorbents often exhibit low CO_2_ adsorption capacity, high costs,^6^ and environmental toxicity and require complex procedures for disposal, making them undesirable for large scale deployment.^6^ Physical adsorption by highly porous materials, such as carbonaceous materials, zeolites, and mesoporous silica, is advantageous because of their low cost, high thermal stability, and high surface area, which enables exceptional uptake of gaseous analytes.^7^ However, these materials are limited by low selectivity and low CO_2_ adsorption capacity.^6^ To address these challenges, the design and synthesis of novel, selective, porous materials derived from earth abundant elements using green chemistry principles remains an active research challenge.

Metal–organic frameworks (MOFs) possess the unique ability to facilitate selective uptake and detection of gases like CO_2_ in complex mixtures (i.e., the atmosphere) due to their permanent porosity enabled through controlled bottom-up assembly via reticular synthesis.^8^ Through tunable modification of pore shape and size by varying the metal nodes and organic linker identity, MOFs function as an efficient system in which to engineer targeted molecular interactions to enable selective uptake and storage of a particular molecule such as CO_2_.^9,10^ Although MOFs offer tremendous promise as materials for uptake and detection of gases,^10−17^ only a limited number of experiments have been developed to emphasize the fundamentals related to MOF synthesis and structure–property relationships at the high school and undergraduate levels.^18−29^ At the undergraduate level, models for experiments encompassing the synthesis and application of MOFs focus on teaching important chemical concepts, such as reticular synthesis,^18−20^ carbon capture and storage (CSS),^21,22^ luminescence, lanthanide chemistry,^23^ and host–guest chemistry.^24,25^ Specifically for cyclodextrin (CD)-based MOFs, there are only two undergraduate laboratory experiments with applications for CO_2_ uptake^26^ and pollutant removal,^27^ and there are even fewer MOF-based experiments for high school students.^26,28^ The experiments developed to date require access to laboratory infrastructure and specialized chemicals that are unsafe and inaccessible in home-based, resource-limited, or other remote-learning settings. The only resource currently available for remote instruction for MOF synthesis and application is video-based and does not offer students hands-on learning opportunities.^29^ To improve equality in the training of the future scientific community, enhance workforce development, and promote global scientific literacy in response to the challenges of remote and hybrid learning emphasized by a global pandemic, development of scientific experimental activities that can be performed without access to a laboratory or specialized chemicals is urgently needed. With MOFs bridging the divide between traditional disciplines of organic and inorganic chemistry, this class of materials provides an excellent opportunity to introduce students to the fundamentals of chemical coordination and porous materials, synthetic techniques such as vapor diffusion for crystal growth, and promote hands-on experience with applications in gas capture and host–guest chemistry.

CD-based MOFs have been proven to not only excel at CO_2_ uptake and storage, but also meet requirements for green synthetic accessibility.^30,31^ The goal of green chemistry is to eliminate or reduce the generation of hazardous substances, while conducting scientific research or while producing materials on the industrial scale.^32,33^ Additionally, green chemistry aims to preserve atom economy, in which all atoms in the starting materials are converted directly to products. The precursors of CD-MOF (gamma-CD (γ-CD), and potassium benzoate (C_7_H_5_KO_2_)) in Figure 1 are renewable raw materials whose degradation process is environmentally compatible. Additionally, the process of MOF crystallization takes place with the use of benign solvents, such as water and ethanol (EtOH), and mild reaction conditions that can be safely carried out in a resource limited setting. This experiment introduces students to fundamentals, such as reticular chemistry, chemical coordination, as well as acid–base and host–guest chemistry, high-value practical skills of synthesis and crystal growth, along with tangible applications of gas capture in a low-resource, remote learning experiment.

**Figure 1:**
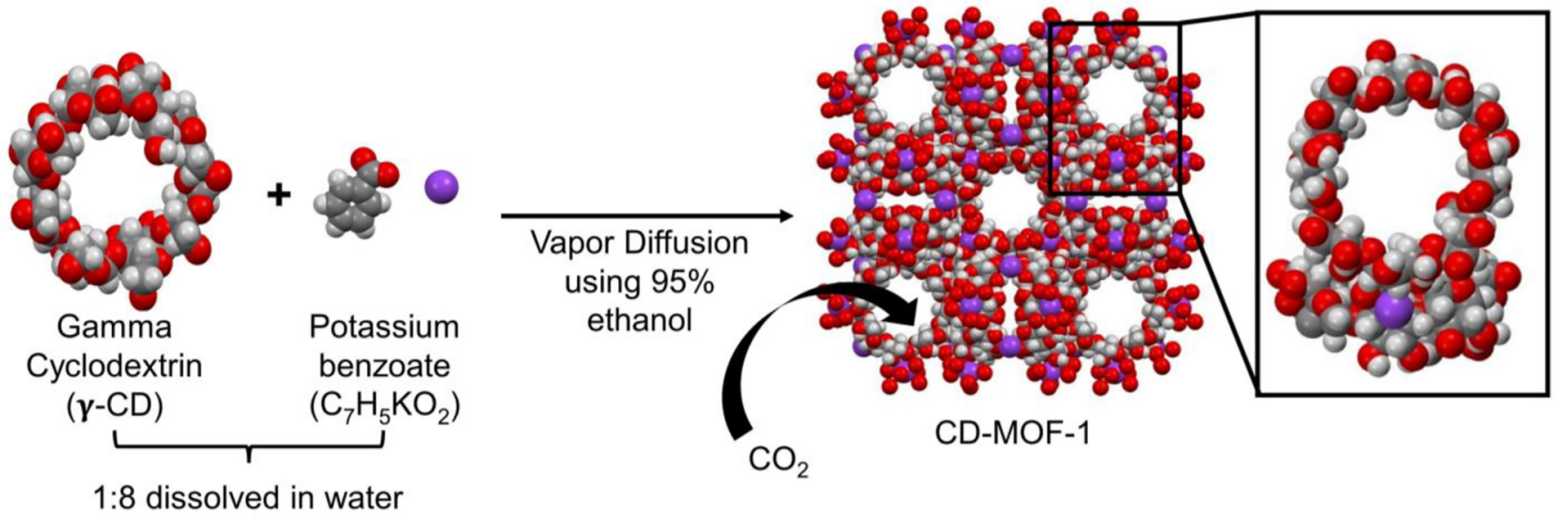
Reaction scheme shows the formation of CD-MOF-1 from reagents γ-CD and C_7_H_5_KO_2_ and the reaction conditions. The crystallographic image of CD-MOF-1 demonstrates CO_2_ uptake into the pores of the structure. To the right is an enhanced image of the coordination between two CD molecules via a potassium atom. Note: The benzoate counterions are not shown in the pores to allow for ease of visualization.

Through the use of a safe and engaging remote experiment, this activity provides students with the instructions to grow a MOF crystal that has been developed through academic research^34^ and gain first-hand experience with important chemical concepts in the context of framework materials: crystal growth, acid–base chemistry, host–guest interactions, and green chemistry principles.^35^ Given the benefits of the kinesthetic learning approach to STEM,^36^ the development of experiments that allow students to physically interact with controlled chemical systems and build meaningful connections to fundamental chemical concepts is an essential feature of future workforce development.^37^

This tailorable remote experiment (see SI section II) describes how high school and undergraduate students can synthesize CD-MOF-1 crystals and qualitatively verify CO_2_ uptake into the pores of the MOF. The experiment was performed remotely by undergraduate students from Dartmouth College (*n* = 4) and as a project in a laboratory setting by high school students from Lebanon High School, NH (*n* = 34). The high school classes consisted of 9 students in a science independent study class and 25 students in an AP Chemistry class. The undergraduate and high school students conducted the experiments in their residences and classrooms, respectively. We adapted the experimental procedure from a previous experimental approach, which was designed to be completed by undergraduates and high school students in a laboratory setting.^26^ The experimental approach described in this paper retains the important intellectual keystones gained in a traditional laboratory setting. Students develop important chemistry skills, such as synthetic dexterity in crystal growth, practical knowledge of crystallization and coordination chemistry, and connections to real-life scientific phenomena of gas uptake by a porous material. The experimental procedure described herein was modified to be safe for remote use, in ways that align with green chemistry protocols, allowing students to draw parallels in regards to health and safety from a small scale up to the macroscopic societal and environmental scales. In this way, students are encouraged to make meaningful connections between how the scientific experiments they perform with their own hands can create potential solutions to global challenges. This experience will introduce students to ideas on how to modify and develop environmentally benign processes and methods in academia and industries. Students are exposed to the idea that sustainable materials must be produced in a sustainable manner, teaching them that green chemistry is not a small, isolated field, but a philosophy applicable to science, technology, and manufacturing.

## Learning Objectives

Learning objectives for the students are divided into three categories: fundamentals, technical skills, and connection of hands-on experience to applied fields and overarching concepts. Through the experimental procedure students explored the principles of green chemistry, reticular chemistry, coordination chemistry, framework materials, acid–base equilibria, and host–guest chemistry. As part of the procedure, students gained synthetic skills such as crystal growth and framework activation. Finally, students used their materials for gas sequestration, orienting their newfound fundamental knowledge and practical skills in real life applications for sustainable solutions to CO_2_ emissions. Prior to experimentation, students and/or facilitators were provided with handouts and videos to familiarize themselves with the basics of the concepts related to the activity (see SI section V).

## Experimental Overview

The preparation and investigation of CD-MOFs can be divided into four sections: (i) rationale for selected reagents and materials, (ii) crystal growth, (iii) crystal activation, and (iv) qualitative CO_2_ uptake analysis. The experimental procedure was adapted from a published laboratory activity for high school students, which required laboratory access, and was modified significantly to enable remote or classroom-based experimentation.^26^ Key modifications included replacing the methanol and dichloromethane (DCM) solvents used for MOF growth with 95% EtOH that is safe for remote experimentation and disposal. C_7_H_5_KO_2_ was used as a MOF precursor instead of potassium hydroxide (KOH) since KOH has a higher acute toxicity while C_7_H_5_KO_2_ can be handled with less precautions and is a common food additive.^38^ Reliance on laboratory equipment was removed by replacing the drying of activated MOF crystals in a high vacuum oven with air-drying the MOF on the countertop at ambient pressure and temperature for 2 days. This modified experiment did not call for the use of an optical microscope, which may not be available, but instructed students to make detailed observations. Additionally, the CO_2_ exposure procedure employed the use of a reaction between baking soda (sodium bicarbonate (NaHCO_3_)) and vinegar (5% acetic acid (5% CH_3_COOH)) instead of sublimation of dry ice.

### Rationale for Selected Reagents and Materials

The experiment was designed so that judicious selection of starting reagents, solvents, and conditions maximized the number of green chemistry principles met and safely facilitated remote implementation. MOFs, framework materials that feature organic molecules coordinated by metal ions, are a well-suited class of materials for this experiment: their porous nature allows for the efficient uptake and storage of gases, while their bottom-up synthetic accessibility enables precise functionalization to tune interactions with guest molecules. The CD-MOF-1 was selected for this procedure because it possesses the ability to be synthesized from the bottom up from natural and benign precursors: γ-CD, a cyclic oligosaccharide, and C_7_H_5_KO_2_, a food-grade salt commonly used as a food additive or preservative.^34^ The use of CD-MOF-1 and the experimental design adheres to the 12 principles of green chemistry.^39^ These benign precursors satisfy the following principles: “Designing Safer Chemicals”, “Use of Renewable Feedstocks”, and “Design for Degradation”. The CD-MOF-1 structure, seen in Figure 1, features repeating motifs of (γ-CD)_6_ that form a body-centered cubic packing arrangement with potassium ions coordinating to the hydroxyl groups on the γ-CD tori to form the cube, as well as to link cubes together.^34^ The use of EtOH to replace the chlorinated solvents satisfies the green chemistry principle of “Safer Solvent & Auxiliaries”. The use of vapor diffusion at room temperature and ambient pressure the following principles are satisfied: “Less Hazardous Chemical Synthesis”, and “Safer Chemistry for Accident Prevention”. MOF activation satisfies “Design for Energy Efficiency” as the crystals are dried under ambient conditions without a vacuum pump and/or vacuum. Finally, the color change of the activated crystals is concomitant to the reaction of NaHCO_3_ and 5% CH_3_COOH, which offers real-time assessment of CO_2_ generation, which satisfies the “Real-Time Analysis for Pollution Prevention” principle. The design of the experiment adheres to 8 of the 12 defined principles of green chemistry and enables students to gain firsthand experience with chemical synthesis and application in a safe manner that informs them of how safety in the microscale of their homes connects to the macroscopic environmental impact.

### Crystal Growth

**Figure 2:**
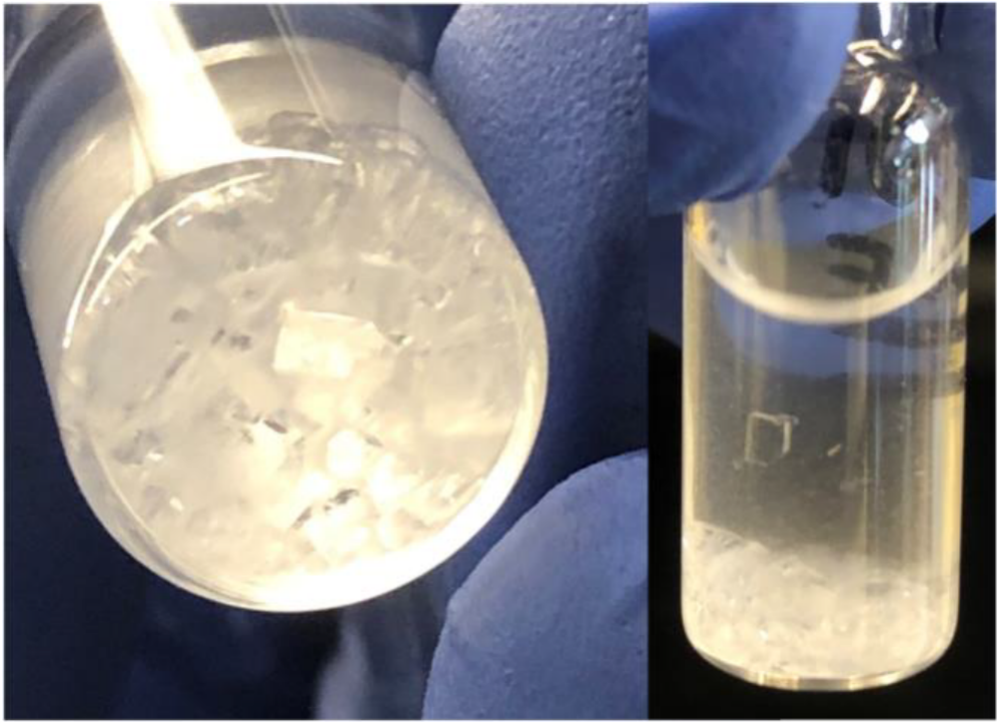
Photographs demonstrate the CD-MOF-1 crystals after 3 days of crystal growth. Note the cubic shape of the crystal and transparent color.

The first step of the experiment is the synthesis and crystal growth of the CD-MOF-1 from food grade γ-CD (0.15 M) and 8 mol equivalent of food grade C_7_H_5_KO_2_ in aqueous solution (see SI section IIa). Crystal growth is achieved by vapor diffusion in which the vapor of a volatile solvent (EtOH) diffuses into the MOF precursor solution, resulting in the crystallization of the MOF due to its insolubility in the volatile solvent. Depending on the availability of materials for students attempting the experiment, different solvents for vapor diffusion may be used. The use of 95% EtOH (available online) (see SI section I) resulted in the largest crystals. While 91% isopropanol (available online and in stores) (see SI section I) afforded smaller cubic crystals, isopropanol is more green in terms of availability and disposal. Crystals took 3–7 days to grow to appreciable sizes of 2 mm in length. There was some variation of crystal growth where some smaller crystals were observed (less than 1 mm in length), but the best results for CO_2_ uptake took place using larger cubic crystals. Students are encouraged to make judgment calls regarding if their crystals require more time to reach a size suitable for CO_2_ uptake (around 2 mm in length) that are optimal for further experimentation. Images of CD-MOF-1 crystals after growth are visible in Figure 2.

### MOF Activation

The second step is the activation of the MOF crystals (see SI section IIb). This process involves evacuation of water molecules and starting reagents from the pores of the CD-MOF-1 to enable optimal CO_2_ uptake as well as incorporating a pH indicator to allow for qualitative analysis. Activation was achieved by removal of the aqueous/alcohol phase from the vial containing the MOF crystals and soaking the crystals in a methyl red indicator solution (1.32 mM) in 95% EtOH. Methyl red is a pH indicator and serves as a qualitative, colorimetric indication of CO_2_ uptake into the pores of the MOF.

The process of soaking the CD-MOF-1 crystals with methyl red solution was completed twice. The CD-MOF-1@methyl red was then soaked in 95% EtOH followed by solvent removal to ensure proper diffusion and evacuation of solvent and uncomplexed indicator from the pores. Next, the crystals were dried by allowing the 95% EtOH within the pores to evaporate in ambient pressure and temperature. The crystals were then covered slightly and left in a cool, dry location for 2 days to enable solvent evaporation from the pores. It is important to note that if the humidity in the location is high, proper solvent evaporation from the pores is diminished and can affect the next steps in the experiment. Drying can be accelerated if humidity is a concern using a vacuum system, or desiccator. If there is no access to this equipment, the drying time can be extended until completed. At the completion of the drying time, the crystals exhibited a yellow color due to incorporation of methyl red into the CD-MOF-1 structure.

Note: While the solvents used in these experiments are not ideal for complete evacuation of the pores due to the small percentage of water (5% for the 95% EtOH and 9% for the 91% isopropanol), this choice was made to accommodate access to household chemicals for remote use. The previously reported procedure^26^ before our modification used hazardous solvents like methanol and DCM that evaporate well from the MOF pores, but are not ideal for remote use and are environmentally deleterious.^11^

### Qualitative CO_2_ Uptake Analysis

CD-MOF-1@methyl red crystals were exposed to CO_2_, and a colorimetric change due to the methyl red pH indicator verified CO_2_ diffusion and uptake in the pores. CO_2_ exposure was achieved via placing the small vial containing activated MOF crystals into a larger vial where a reaction between 0.4 g of NaHCO_3_ and 5 mL of 5% CH_3_COOH took place. NaHCO_3_ and 5% CH_3_COOH first react via a double displacement in which the ions exchange to form sodium acetate (CH_3_COONa) and carbonic acid (H_2_CO_3_). Once the H_2_CO_3_ is formed, it decomposes to form water and CO_2_, which is acidic. The gaseous CO_2_ then diffuses throughout the experimental setup and is sequestered by CD-MOF-1@methyl red by adsorption onto the framework.^40^

**Figure 3:**
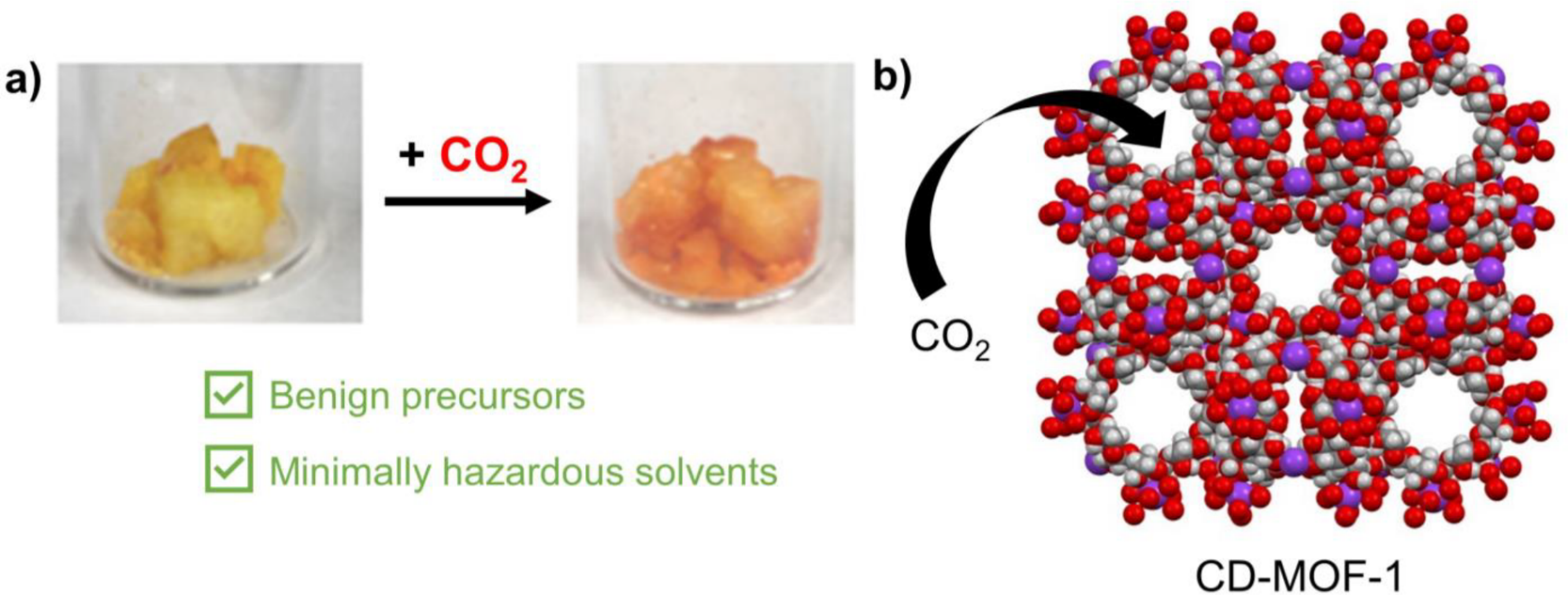
(a) Images of CD-MOF-1 with methyl red indicator before and after CO_2_ exposure demonstrating analyte uptake qualitatively. (b) Pictorial representation of CO_2_ uptake by CD-MOF-1.

Qualitative colorimetric CO_2_ detection is enabled by the methyl red indicator. The CD-MOF-1@methyl red crystals are yellow before exposure and turn red when exposed to acidic CO_2_ as seen in Figure 3. The observed color change is reversible after a minimum of 20 min as the CO_2_ interacts with the material via an adsorption–desorption process.

## Hazards

Students should complete the experiment under the supervision of a guardian or an instructor. Both student and guardian/instructor should carefully read safety instructions. All experimenters should wear proper personal protective equipment (safety glasses, gloves, long sleeves and pants, and closed-toed shoes).

γ-CD and methyl red pose minimal hazards, but proper precautions should be taken to avoid inhalation, ingestion, or contact with skin or eyes. Solvents like 95% EtOH and 91% isopropanol are flammable and should be stored in a cool, dry place and used in a well-ventilated environment and away from open flames. For proper disposal procedures of materials and waste generated in the experimental procedure, see SI section VI instructions.

## Results

**Figure 4:**
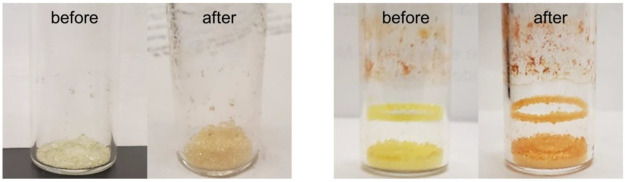
Images collected by two high school students from Lebanon High School depicting qualitative data collected from two CO_2_ uptake experiments. The color of activated CD-MOF-1@methyl red crystals is documented before and after exposure to CO_2_. The change in color from yellow to orange indicates CO_2_ uptake by the MOF.

The MOF synthesis and formation has been carried out by 4 undergraduate students and 34 high school students in two classes, a science independent study class and AP Chemistry. All experimenters grew crystals of CD-MOFs over the course of 3–7 days. The students in the independent study (*n* = 9) successfully completed the experiment and digitally recorded the color change of the CD-MOF-1@methyl red when exposed to CO_2_ via a reaction between 0.4 g of NaHCO_3_ and 5% CH_3_COOH as seen in Figure 4. The students in the AP Chemistry (*n* = 25) class successfully grew CD-MOF-1 crystals.

Based on student reflections, they gained valuable laboratory skills and were exposed to new areas of chemistry and research. One student wrote, “*This experiment has allowed me to make connections between the concepts I have learned and their real-world applications... I also learned that reticular synthesis is the building up of complex network structures like MOFs and other crystals, and through the process of vapor diffusion, extremely complex structures can be formed if the process happens slowly enough... Due to their structure, MOFs have the capacity to “trap” various different gases and molecules. They are made up of a network of organic material held together by metal particles. MOFs have a number of interstices in which large amounts of particles can be contained in a relatively small volume*.” 4 undergraduates from Dartmouth College also completed all steps of the experiment successfully in a remote setting. This experiment enabled them to engage in scientific experimentation while laboratory access was limited due to COVID-19 restrictions. Students both performed the experiment and interacted with the topics thoughtfully.

The synthetic procedure that resulted in the most optimal CD-MOF-1 growth and minimization of CD precipitation involved 95% EtOH vapor diffusion into a 1:8 aqueous solution of γ-CD:C_7_H_5_KO_2_. Under these conditions cubic crystals began forming after 12–24 h, and after 3 days the crystals averaged ∼2 mm in size. Full descriptions of the synthetic procedure along with photographs detailing each step are provided in SI section V.

**Figure 5:**
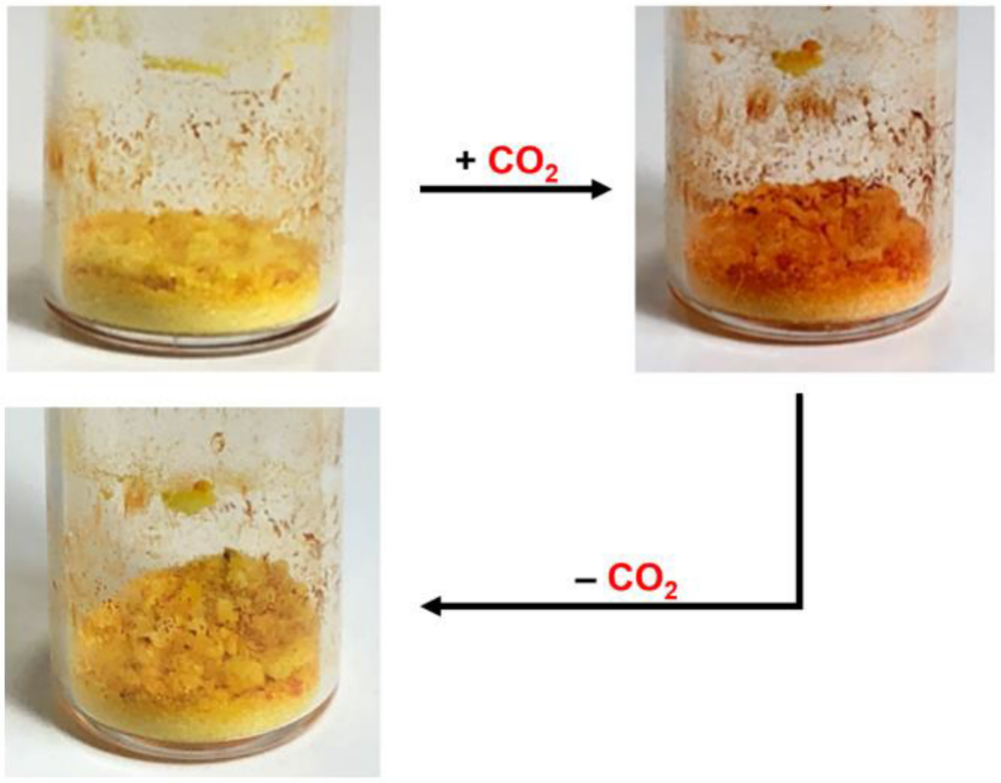
Reversible adsorption and desorption of CO_2_ resulting in the visible color change in the CD-MOF-1@methyl red crystals. Images were taken before CO_2_ exposure, immediately after CO_2_ exposure, and 20 min after CO_2_ exposure result in a color change from yellow, to orange, and back to yellow, respectively.

Figures 4 and 5 show the images of the MOF color change resulting from exposing CD-MOF-1 to CO_2_ evolving from the NaHCO_3_ and 5% CH_3_COOH reaction. The generation of CO_2_ from NaHCO_3_ and 5% CH_3_COOH reaction allowed for diffusion of gaseous CO_2_ into the pores of the MOF. The colorimetric change of CD-MOF-1 with methyl red incorporated in its pores has been successful in the hands of a graduate student (*n* = 1) and undergraduate (*n* = 4) and is catalogued in Figure S4. This section of the experiment was also completed by some of the participating high school students (*n* = 9). Reversible sorption of CO_2_ by the methyl red-activated CD-MOF-1 was observed as shown in Figure 5. After CO_2_ exposure via the reaction between 5 mL of 5% CH_3_COOH and 0.4 g of NaHCO_3_, the methyl red-activated MOF color was orange indicating CO_2_ sorption. Methyl red exhibits a yellow color when in an environment with a pH over 6.2 and a red color when in an environment under 4.4. In 4.4 < pH < 6.2, methyl red exhibits an orange color. When the MOF structure is exposed to CO_2_, the methyl red deepens its color to a reddish hue, hereby indicating CO_2_ uptake by the MOF. This colorimetric change indicates the adsorption of CO_2_ into the framework. Over the course of 20 min, the CO_2_ desorbed from the MOF framework to result in a color change from orange back to yellow, indicating that CO_2_ was no longer present.

## Summary

Green chemistry draws a natural parallel between remote experimentation and environmental sustainability; at-home experiments must fulfill green chemistry principles to be safe for students just as large scale processes in industries must adhere to the same principles to protect the environment and all individuals living in it. This activity guides students in synthesizing and activating CD-MOF-1 crystals as well as using them as materials for gas uptake and storage. In this experiment students explore crystal growth through the vapor diffusion synthetic portion, acid–base chemistry through using methyl red as an indicator, and host–guest chemistry through the qualitative CO_2_ uptake section. Students also gain understanding of fundamental concepts related to green chemistry through exploring why the judicious selection of benign MOF precursors and solvents is imperative.

The experiment described herein provides students with the opportunity for hands-on experience synthesizing framework materials to be used as a solution for the relevant issues negatively impacting our society such as greenhouse gas emissions and their subsequent effect on climate change. Equally important, students address this issue using green, and relatively benign, precursors and methods to produce a sustainable solution. Students are not simply provided facts of our collective reality but are empowered to synthesize and work with materials used in professional science to address societal challenges through green chemistry solutions.

## Supporting Information Available

Instructor notes including student handouts (PDF)
Slides for instructor presentation (PDF)
an instructional video detailing fundamental concepts and procedural steps (MP4)
