# Supplementary material for: CD-MOF-1 for CO2 Uptake: Remote and Hybrid Green Chemistry Synthesis of a Framework Material with Environmentally Conscious Applications
Source: J Chem Educ. 2023 Feb 13;100(3):1289–95. doi: 10.1021/acs.jchemed.2c00922 (PMC10018730; doi:10.1021/acs.jchemed.2c00922)
Supplement: Supplementary file 1 — ed2c00922_si_001.pdf [file ed2c00922_si_001.pdf]

CD-MOF-1 for CO_2 Uptake: Remote and Hybrid Green Chemistry Synthesis of a Framework Material with Environmentally Conscious Applications

Georganna Benedetto[1], Brittany M. Cleary[1], Colin T. Morrell[1], Claudia G. Durbin[1], Anna L. Brinks[1], John Tietjen[2], and Katherine A. Mirica[1*]

[1]Department of Chemistry, Dartmouth College, Burke Laboratory, 41 College St., Hanover, NH 03755, USA.

[2]Lebanon High School, 195 Hanover St., Lebanon, NH 03766, USA.

*Email: Katherine.A.Mirica@dartmouth.edu

SUPPORTING INFORMATION

	**Section**	**Page**
	Contents	S1
I.	Materials and Supplies	S2
II.	Methods	S5
III	Results	S11
IV	CD-MOF-1 Identification	S15
V	Student Handouts	S18
VI	Safety Instructions	S40
VII	High School Student Results	S41
VIII	Undergraduate Research Reports	S42
IX	References	S42

I. Materials and Supplies

The following sections are divided into two (**a. Kit Provided Procedure** and **b. Independent Experiment**). The 4 undergraduate students who received an experimental kit were provided with enough chemicals and materials packaged to conduct triplicate trials of the experiment. The undergraduate students picked up the kits from Burke Laboratories at Dartmouth and they returned the kits after the experiment was conducted for proper hazardous waste disposal. The chemicals were listed with their accompanying vial name. For those who did not receive an experimental kit but were interested in conducting the experiment, the chemicals, supplies, as well as suppliers and prices were included.

To streamline the laboratory procedure, the instructor may choose to create kits for students to work with.

a. Provided in Kit

I. Materials and Supplies (per 1 experiment)

i. Chemicals

1. 195 mg gamma cyclodextrin (γ-$C_{48}H_{80}O_{40}$) in a 20 mL glass vial labeled **"Gamma Cyclodextrin (γ-CD)"**
2. 192.4 mg potassium benzoate ($C_7H_5KO_2$) in a 20 mL plastic vial labeled **"Potassium Benzoate"**
3. 4 mL 95% ethanol (EtOH) in a 20 mL plastic vial labelled **"4 mL EtOH"**
4. 1 mL deionized water (DI H_2O) in a 20 mL plastic vial labelled **"1 mL DI H_2O"**
5. 7.7 mg of methyl red sodium salt labelled **"7.7 mg methyl red"** and 20 mL 95% EtOH labelled **"20 mL EtOH"** to make 1.32 mM stock solution

6. 0.4 g sodium bicarbonate ($NaHCO_3$) in a 20 mL plastic vial labeled **"0.4 g $NaHCO_3$"**
7. 5 mL white vinegar (5% acetic acid (5% CH_3COOH)) in a 20 mL plastic vial labeled **"5 mL 5% CH_3COOH"**

ii. Supplies

1. 3 mL syringe (one)
2. Cotton ball (one)
3. Forceps
4. 1 (one) 20 mL scintillation vial
5. 1 (one) 1.8-dram vials
6. 3 mL disposable plastic pipettes (five)
7. Safety glasses
8. Nitrile Gloves

Note: When instructors are assembling kits it is possible to add safe silica gel packets to control for high humidity locations during kit storage and delivery. (Desiccant available for purchase on Amazon.com.)

b. Independent Experiment

i. Chemicals from Manufacturers

1. Food grade gamma cyclodextrin (γ-$C_{48}H_{80}O_{40}$) (TGCD-F) was sourced from CTD, Inc. (Alachua, FL)
2. Potassium benzoate ($C_7H_5KO_2$) (EG-C7H5KO2-4) was sourced from Eisen-Golden Laboratories and purchased on ebay.com for $8.99

3. 95% ethanol (UN1170) was sourced from Home Science Tools (Billings, MT) ($5.35/ 30mL)
4. Deionized water (4 mL) in vial labelled "**DI H_2O**"
5. Methyl red, sodium salt ($C_{15}H_{14}N_3NaO_2$) (845-10-3) was sourced from Fisher Science Education ($11.25/10g)

ii. **Chemicals Available for Purchase in Grocery Stores**

1. Sodium bicarbonate (Baking soda ($NaHCO_3$))
2. White vinegar (5% acetic acid (5% CH_3COOH))

iii. **Supplies from Manufacturers**

1. Scale (AMIR Digital Kitchen Scale, 500 g)
2. 3 mL NORM-JECT syringe (one)
3. Forceps
4. 20 mL scintillation vial with cap (four)
5. 1.8-dram vial with cap (one)
6. Pipettes (five) (plastic)
7. Safety glasses
8. Nitrile gloves

iv. **Supplies Available for Purchase in Stores**

1. Cotton ball (one)

II. Methods

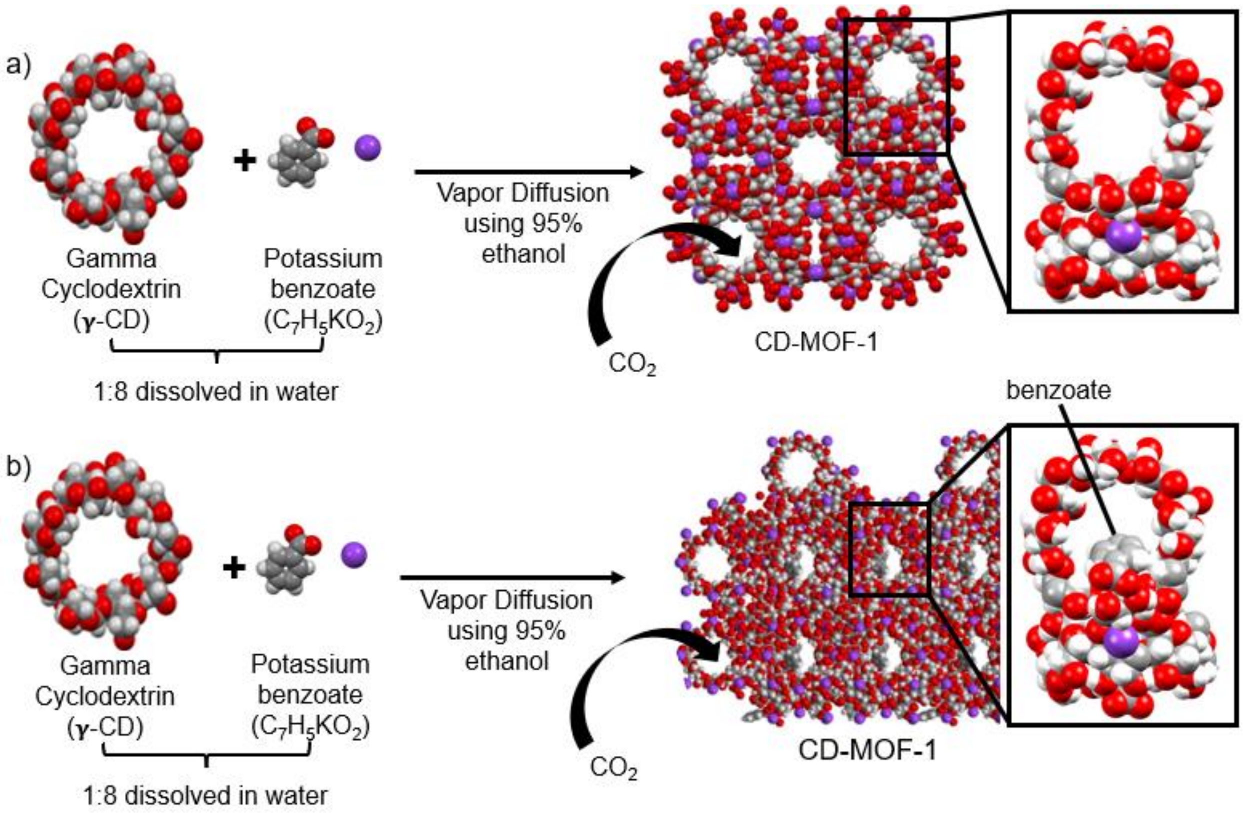

Figure S1: The reaction scheme shows the formation of CD-MOF-1 from reagents γ-CD, and $C_7H_5KO_2$ with reaction conditions. To the right is an enhanced image of the coordination between two CD molecules via a potassium atom. (b) The same reaction scheme as in a) is shown but the MOF is depicted with benzoate counterions in the pores of the MOF. As opposed to figure S1a, this scheme shows the benzoate counterions in the pores of the MOF.

a. Optimized Procedure for CD-MOF-1 Crystal Growth by Vapor Diffusion:

i) Kit Provided Procedure

1. Proper PPE was put on and chemicals #1–4 and supplies #1–5 were collected.
2. Contents were transferred from vial labelled "**Potassium Salt**" (192.4 mg) to the "**γ-CD**" (195 mg) vial to combine the potassium benzoate and gamma cyclodextrin precursors of the MOF.

3. 1 mL of deionized water from vial labelled **“1 mL DI H_2O”** was poured into “**γ-CD**” vial and the vial was capped and gently swirled to facilitate dissolving of the precursors. It took a few minutes to fully dissolve.
4. 1 mL of MOF reagent solution was drawn up into a 3 mL syringe.
5. A filter was formed from a small piece of a cotton ball by rolling it between the pointer finger and thumb.
6. To enable filtering of the solution before its transfer to the 1.8 mL vial, the syringe was inverted so the nozzle was solution free. A small piece of a cotton ball was placed in the nozzle to serve as a filter.
7. The solution in the syringe was filtered into a 1.8 mL dram vial. (Note: plunger was pressed gently so that the cotton filter does not dislodge.)
8. 4 mL of 95% ethanol from the vial labelled **“4 mL EtOH 95%**” was added to an empty 20 mL scintillation vial.
9. Using tweezers (small tongs would work as well) the 1.8 mL vial with MOF reagents was carefully transferred into the 20 mL scintillation vial so that the meniscus of the ethanol was above the meniscus of the inner vial.
10. The outer vial was capped tightly. (Note: set up was moved carefully so that the inner vial does not tip).
11. The experimental set up was left undisturbed in a dark location for 3−7 days for vapor diffusion to allow for proper crystal growth. The vial was observed day to day to monitor crystal growth. (Note: white powder was the cyclodextrin precipitating of solution. Cubic, clear larger crystals were the desired CD-MOF-1.)

ii) Independent Experiment Procedure:

1. Proper PPE was put on and chemicals #1–4 from manufacturers, supplies form manufacturers #1–5, and supply from stores #1.
2. 390 mg of γ-CD and 384.8 mg of potassium benzoate ($C_7H_5KO_2$) were massed and dissolved in 2 mL of DI water.
3. 1 mL of MOF reagent solution was drawn up into a 3 mL syringe.
4. A filter was formed from a small piece of a cotton ball by rolling it between the pointer finger and thumb.
5. To enable filtering of the solution before its transfer to the 1.8 mL vial, the syringe was inverted so the nozzle was solution free. A small piece of a cotton ball was placed in the nozzle to serve as a filter.
6. The solution in the syringe was filtered into a 1.8 mL dram vial. (Note: plunger was pressed gently so that the cotton filter does not dislodge.)
7. A 20 mL scintillation vial was filled with 4 mL 95% ethanol.
8. Using tweezers (small tongs would work as well) the 1.8 mL vial with MOF reagent solution was carefully transferred into the 20 mL scintillation vial so that the meniscus of the ethanol was above the meniscus of the inner vial.
9. The outer vial was capped tightly. (Note: set up was moved carefully so that the inner vial does not tip).
10. The experimental set up was left undisturbed in a dark location for 3−7 days for vapor diffusion to allow for proper crystal growth. The vial was observed day to day to monitor crystal growth. (Note: white powder was the cyclodextrin precipitating out of solution. Cubic, clear larger crystals were the desired CD-MOF-1.)

b. Activation of CD-MOF-1 and Indicator Incorporation:

i) Kit Provided Procedure:

1. Proper PPE was put on and chemical #5 and supply #6 was collected.
2. The inner vial containing crystals was removed from the larger vial and dried using a paper towel. The solvent (now DI water and alcohol due to vapor diffusion) in the inner vial surrounding the crystals was pipetted out and disposed.
3. The contents of vial **"20 mL EtOH 2"** was poured into the vial labelled **"7.7 mg methyl red"** to afford a 1.32 mmol solution of methyl red sodium salt indicator in 95% ethanol. To help the methyl red dissolve, a pipette was used to agitate the solution.
4. A couple mLs of methyl red indicator solution was pipetted into small vial with MOF crystals using a 3 mL plastic disposable pipette so that all the crystals were completely submerged.
5. The crystals were left soaking for 1–24 hours to allow for proper diffusion. Over the course of the diffusion, it was possible to see the crystals change from slightly transparent to yellow.
6. After 1–24 hours, the methyl red indicator solution was pipette out and disposed of. The crystals looked light yellow. (Note: If the crystals did not look yellow, the time for solvent exchange was increased by leaving the crystals submerged in the methyl red solution for longer.)
7. Steps **4–6** were repeated.
8. Pure 95% ethanol was pipetted into the small vial and left for 1–24 hours. After 1–24 hours, the ethanol was pipette out and disposed of. This step is to complete the

activation of the MOF and evacuate the rest of the water from the pores of the MOF.

9. The small vial of MOF crystals was lightly capped and left for 2 days to dry. The final activated crystals look yellow. If left uncapped for a long time, the edges of the crystals darkened to a deeper orange, which we want to avoid.

   Note: Drying can be accelerated if humidity is a concern using a vacuum system, or desiccator. If there is no access to this equipment, the drying time can be extended until completed.

ii) Independent Procedure:

1. Proper PPE was put on and chemical #3 & #5 and supply #6 were collected.
2. The inner vial containing crystals was removed from the larger vial. The solvent (now DI water and alcohol due to vapor diffusion) in the inner vial surrounding the crystals was pipetted out and disposed of
3. A 1.32 mM solution of methyl red indicator in 95% ethanol was made by dissolving 7.7 mg of methyl red sodium salt in 20 mL of 95% ethanol. To help the methyl red dissolve, a pipette was used to agitate the solution.
4. The methyl red indicator solution was pipetted into small vial so that all the MOF crystals were completely submerged.
5. Vial was capped and MOF crystals were left soaking for 1–24 hours to allow for proper diffusion. Over the course of the diffusion, it was possible to see the crystals change from slightly transparent to yellow. (Note: If the crystals did not look yellow, the time for solvent exchange was increased by leaving the crystals submerged in the methyl red solution for longer.)

6. After 1–24 hours, the methyl red indicator solution was pipette out and disposed of. The crystals looked light yellow.
7. Steps **4–6** were repeated.
8. 95% ethanol was pipetted into the small vial and left for 1–24 hours. After 1–24 hours, the ethanol was pipette out and disposed of. This step was performed to complete the activation of the MOF and evacuate the rest of the water from the pores of the MOF.
9. The small vial of MOF crystals was lightly capped and left for 2 days to dry. The final activated crystals look yellow. If left uncapped for a long time, the edges of the crystals darkened to a deeper orange.

   Note: Drying can be accelerated if humidity is a concern using a vacuum system, or desiccator. If there is no access to this equipment, the drying time can be extended until completed.

c. Colorimetric Analysis of CO_2 Uptake by CD-MOF-1:

i) Kit Provided Procedure:

1. Proper PPE was put on and chemical #6–7 and supply #6 were collected.
2. The color of the CD-MOF-1 crystals before CO_2 exposure was noted.
3. The small vial with activated CD-MOF-1@methyl red crystals was uncapped. The vial was added to the larger vial labeled "**Sodium bicarbonate**" using tweezers or tongs.
4. Using a pipette, 5 mL of white vinegar (in a vial labeled "**5% acetic acid**") was added to the outer vial labelled "**Sodium bicarbonate**". The vinegar was added in two aliquots so that the reaction did not bubble into the small vial.

5. The vial was lightly capped once all the vinegar was added. The MOF crystal color change was observed as the vinegar-baking soda reaction progressed and produced CO_2.
6. Once the vinegar-baking soda reaction ceased, the inner vial with CD-MOF-1@methyl red crystals was removed to better observe the color change.
7. The CD-MOF-1@methyl red crystals were left uncapped for about 20 minutes to enable CO_2 desorption. The MOF color change back to yellow was observed.

ii) Independent Procedure:

1. Proper PPE was put on and chemical from the store #1–2 and supplies #4 & #6 were collected.
2. 0.4 g of baking soda was added to a 20 mL scintillation vial and the small vial with activated MOF was placed inside using tweezers.
3. Using a pipette, 5 mL of white vinegar was added to the outer vial with the baking soda. The vinegar was added in two aliquots so that the reaction did not bubble into the small vial.
4. The vial was lightly capped once all the vinegar was added. The MOF crystal color change was observed as the vinegar-baking soda reaction progressed and produced CO_2.
5. Once the vinegar-baking soda reaction ceased, the inner vial with CD-MOF-1@methyl red crystals was removed to better observe the color change.
6. The CD-MOF-1@methyl red crystals were left uncapped for about 20 minutes to enable CO_2 desorption. The MOF color change back to yellow was observed.

III. Results

i) Trials working towards an optimized procedure for CD-MOF-1 Crystal Growth by Vapor Diffusion

Trial	MOF reagent solution (MS)	Crystallization Conditions	Observations	Activation (yes/no)	CO_2 exposure (yes/no)
1	778 mg λ-CD (A)<br>492 mg $C_7H_5KO_2$ (B)<br>DI H_2O	0.75 mL MS<br>91% isopropanol<br>5 days	powdery with some larger crystals around lid	Yes	Yes
2	778 mg A<br>492 mg B<br>DI H_2O	0.75 mL MS<br>95% ethanol<br>5 days	powdery with some larger crystals around lid	Yes	Yes
3	390 mg A<br>50 mg B<br>DI H_2O (2 mL)	0.75 mL MS<br>95% ethanol<br>3 days	cubic larger crystals- PXRD- confirm	Yes	Yes
4	390 mg A<br>50 mg B<br>DI H_2O (2 mL)	0.75 mL MS<br>91% isopropanol<br>3 days	powder	No	No
5	390 mg A<br>50 mg B<br>DI H_2O (2 mL)	0.1 mL MS<br>91% isopropanol<br>3 days	small cubic crystals	Yes	Yes
6	975 mg A<br>125 mg B<br>DI H_2O (5 mL)	0.75 mL MS<br>95% ethanol<br>2 weeks	@3 days: no crystals, @ 2 weeks: large crystal on side (cubic crystals and powder	Yes (w/ methyl red)	Yes
7	975 mg A<br>125 mg B<br>DI H_2O (5 mL)	0.25 mL MS<br>95% ethanol<br>2 weeks	no crystals after 3 days, left for two weeks and cubic crystals in white powder grew	Yes (w/ methyl red)	Yes
8	975 mg A<br>125 mg B<br>DI H_2O (5 mL)	4 mL MS*<br>91% isopropanol<br>2 weeks	lots of white powder	No	No
9	390 mg A<br>50 mg B<br>DI H_2O (2 mL)	0.75 mL MS<br>95% ethanol<br>1 week	cubic crystals with some powder	Yes (w/ methyl red)	No
10	390 mg A<br>50 mg B<br>DI H_2O (2 mL)	0.75 mL MS<br>95% ethanol<br>1 week	cubic crystals with some powder	Yes (w/ methyl red)	No
11	390 mg A<br>50 mg B<br>DI H_2O (2 mL)	0.75 mL MS<br>91% isopropanol<br>1 week	smaller crystals with powder	No (w/ methyl red)	No
12	1: 8 A: B<br>DI H_2O (2 mL)	1 mL MS<br>95% ethanol<br>(4 mL) Aldon-Amazon<br>3 days	Large cubic crystals (no white powder)	Yes (w/out methyl red	No
13	1: 8 A: B<br>DI H_2O (2 mL)	1 mL MS<br>95% ethanol<br>(8 mL)<br>3 days	Large cubic crystals (no white powder)	Yes (w/out methyl red	No

14	1: 8 β-CD: B<br>DI H_2O (2 mL)	1 mL MS<br>95% ethanol<br>(4 mL)<br>3 days	No crystals	N/A	No
15	1: 8 β-CD: B<br>DI H_2O (2 mL)	1 mL MS<br>95% ethanol<br>(8 mL)<br>3 days	No crystals	N/A	No
16	0.390 g A<br>0.385 g B<br>(1:8 γ-CD:B)<br>2 mL DI H_2O	1 mL MS<br>8 mL 95% ethanol	Small to medium sized cubic crystals with some white powder	Yes<br>(w/methyl red)	Yes
17	0.390 g A<br>0.385 g B<br>(1:8 γ-CD:B)<br>2 mL DI H_2O	1 mL MS<br>8 mL 95% ethanol	Medium sized cubic crystals	Yes<br>(w/methyl red)	Yes
18	0.390 g A<br>0.385 g B<br>(1:8 γ-CD:B)<br>2 mL DI H_2O	1 mL MS<br>8 mL 95% ethanol	Small to medium sized cubic crystals with some white powder	Yes<br>(w/methyl red)	No
19	0.390 g A<br>0.385 g B<br>(1:8 γ-CD:B)<br>2 mL DI H_2O	1 mL MS<br>8 mL 95% ethanol	Small to medium sized cubic crystals with some white powder	Yes<br>(w/methyl red)	No
20	0.390 g A<br>0.385 g B<br>(1:8 γ-CD:B)<br>2 mL DI H_2O	1 mL MS<br>8 mL 95% ethanol	Small to medium sized cubic crystals with some white powder	Yes<br>(w/methyl red)	No
21	0.390 g A<br>0.385 mg B<br>(1:8 γ-CD:B)<br>2 mL DI H_2O	1 mL MS<br>8 mL 95% ethanol	Small to medium sized cubic crystals with some white powder	Yes<br>(w/methyl red)	No
22	50 mg A<br>50 mg B<br>2 mL DI H_2O	0.75 mL MS<br>8 mL 95% ethanol	Medium sized cubic crystals	No	No
23	200 mg A<br>200 mg B<br>3 mL DI H_2O	1 mL MS<br>8 mL 95% ethanol<br>(Graves Grain Alcohol)	Large cubic crystals	Yes<br>(w/methyl red)	Yes
24	0.39 g A<br>0.38 g B<br>2 mL DI H_2O	1 mL MS<br>8 mL 95% ethanol	Small cubic crystals	Yes<br>(w/methyl red)	Yes
25	0.39 g A<br>0.38 g B<br>2 mL DI H_2O	1 mL MS<br>4mL 95% ethanol	Small cubic crystals	Yes<br>(w/methyl red)	Yes
26	0.78 g A<br>0.715 g B<br>4 mL DI H_2O	1 mL MS<br>4 mL 95% ethanol	Small cubic crystals	Yes<br>(w/methyl red)	Yes
27	0.78 g A<br>0.715 g B<br>4 mL DI H_2O	1 mL MS<br>6 mL 95% ethanol	Smaller cubic crystals than trial 26	Yes<br>(w/methyl red)	Yes
28	0.78 g A<br>0.715 g B<br>4 mL DI H_2O	1 mL MS<br>8 mL 95% ethanol	Small cubic crystals than trials 26 and 27	Yes<br>(w/methyl red)	Yes

Table S1: Synthetic conditions attempted for MOF crystallization via vapor diffusion.

ii) Results for Activation of CD-MOF-1 and Indicator Incorporation:

Trial	Conditions	Methyl Red (yes/no)	Molarity Methyl Red (mM) with solvent

1	DCM (1x), Ethyl Acetate (2x) 1 hr each.<br>dry in vacuum oven (120 ^{o}C) 1 hr	No	---
2	DCM (1x), Acetone (2x) 1 hr each.<br>dry in vacuum oven (120 ^{o}C) 1 hr	No	---
3	Acetone (3x) 1 hr each.<br>dry in vacuum oven (120 ^{o}C) 1 hr	No	---
5	Acetone (3x) 1 hr each.<br>dry in vacuum oven (120 ^{o}C) 2 hr	No	---
6	Methyl Red Solution (3x), 1 hr each, dry in ambient air conditions (3 days)	Yes	0.927<br>95% ethanol
7	Methyl Red Solution (3x), 1 hr each, dry in ambient air conditions (3 days)	Yes	0.927<br>95% ethanol
9	Methyl Red Solution (3x), (2 hr, 2 hr, ~12 hr), DCM (1x) 1 hr, dry in ambient air conditions (3 days)	Yes	0.927<br>DCM
10	Methyl Red Solution (3x), (2 hr, 2 hr, ~12 hr), DCM (1x) 1 hr, dry in ambient air conditions (3 days)	Yes	0.927<br>DCM
11	Per vial (6 trials): (2x) 2 mL of Methyl Red Solution, 2 mL ethanol (24 hrs), (1x) 4 mL ethanol (24 hrs), dried in ambient air (24 hrs) (fine white sediment removed with each wash, until ethanol wash supernatant was mostly free of material, but yellow in color)	Yes	1.32<br>95% ethanol
12<br>(for trials 26-28 from Table S1)	Methyl Red Solution (2x), (8 hr, 12 hr), 95% ethanol (1x) (8 hrs)<br>Dry in ambient air conditions (1 day)	Yes	8.23 mM<br>95% ethanol

Table S2: Activation condition trials.

iii) CO_2 Uptake Analysis: Dry Ice Method

Trial	Methyl Red (yes/no)	Color change	mass crystals + vial (g)	mass crystals + vial after CO_2 uptake (g)	CO_2 uptake (g)
1	No	N/A	1.96834	1.9712	0.00286
2	No	N/A	1.97481	1.97544	0.00063
3	No	N/A	2.14325	2.151	0.00775
5	No	N/A	1.96896	1.97134	0.00238
6	Yes	Yellow to orange	--	--	--
7	Yes	Yellow to orange	--	--	--

Table S3: Trials of optimizing qualitative and quantitative data for CO_2 uptake. Note: all experimental set ups were as follows: small bit of dry ice (~1 g) was added to 20 mL scintillation vial, small vial with activated CD-MOF-1 crystals were added into scintillation vial with dry ice, outer vial was lightly capped, sublimation occurred for 10 minutes before removal of crystals for analysis)

iv) CO_2 Uptake Analysis: Baking Soda and Vinegar Method

Trial	Color before CO_2 exposure	Baking Soda Amount (g)	Vinegar Amount (mL)	Description of Set Up	Color after CO_2 exposure

1	Orange	0.08	1	Amounts were calculated to yield 20 mL of CO_2. Baking soda and small vial with activated MOF was placed inside 20 mL scintillation vial. Vinegar added with syringe and vial capped quickly.	No noticeable change[a]
2	Orange	0.4	5	Baking soda was added to a 50 mL flask and covered with saran wrap. A straw was used to connect the flask to the small vial with activated MOF. Vinegar was added and then saran wrap was replaced quickly.	No noticeable change[a]
3	Orange	excess	excess	Same as Trail 2 but vinegar added slowly to see if exposure time is an issue.	No noticeable change[a]
4	Yellow	0.084	1	Baking soda and uncapped vial containing CD MOF placed into 20 mL scintillation vial. Vinegar added using a plastic pipette. Vial capped quickly. Gradual color change of MOF from yellow to orange over approximately 7.5 minutes.	Yes<br><br>Starting mass (vial + MOF): 5.172 g<br><br>Mass after uptake: 5.176 g<br><br>Mass CO2: 0.004 g
5	Yellow	1	6	Adapted from 2. A 20 mL scintillation vial was used instead of a 50 mL flask due to what was available.	No noticeable change. Mass remained 5.242 g. Issue with saran wrap coming off during trial
**6**[b]	Yellow	0.4	5 mL	**Optimized:** See Procedure **ci** in **Section SI II**	Color changed to red/orange
**7**[b]	Yellow	0.4	5 mL	See Procedure **ci** in **Section SI II**	Color changed to red/orange
**8**[b]	Yellow	0.4	5 mL	See Procedure **ci** in **Section SI II**	Color changed to red/orange

Table S4: Trials of optimizing CO_2 uptake by CD-MOF-1 crystal with qualitative data. CO_2 is generated from a baking soda-vinegar reaction. [a]Trials 1−3 resulted in no noticeable color change. This is most likely because the MOF crystals had been left exposed to the atmosphere for up to 7 days after being activated with methyl red. The crystals were orange instead of yellow. [b]Trials 6−8 resulted in noticeable color change and were done using the optimized procedure detailed in section **ci.**

IV. CD-MOF-1 Identification

Framework solids that are periodic form a structure called a crystal structure that can be classified by the particular way the atoms arrange themselves.[2] For instance, table salt (NaCl) forms a cubic structure where the larger chloride ions pack cubically and the smaller sodium ions reside periodically in the voids. An interesting phenomenon is that the molecular structure of the components can determine the larger macroscopic shape of the crystal. For instance, the hexagonal shape iconic to snowflakes arises because water molecules coordinate in hexagonal patters at the molecular level when frozen.

In a laboratory setting, crystals such as CD-MOF-1 can be identified by a technique called powder X-ray diffraction (PXRD). The material is placed in the instrument and is irradiated with X-rays. The material scatters the X-rays in a process called diffraction. The distances between atoms in a framework structure can be resolved by PXRD because these distances are on the same length scale as the X-rays that are irradiating the sample. The different space groups of CD-MOF-1 are seen in **Figure S2**. Based on the intensities (y axis) and scattering angle (x axis) of the graph seen in **Figure S3**, the structure of CD-MOF-1 can be deconvoluted.

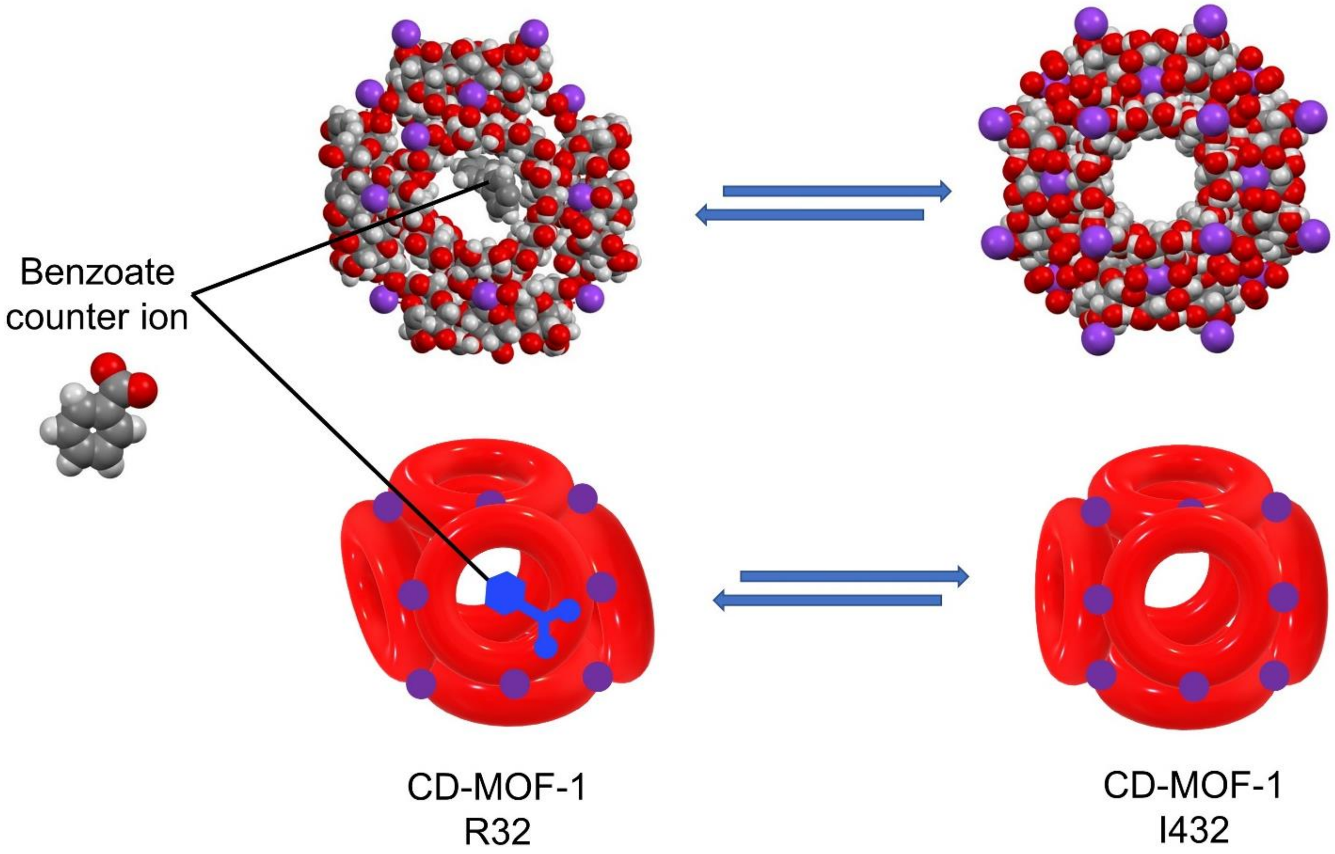

Figure S2: CD-MOF-1 ion exchange transformation.

For the purposes of at home experimentation, the visual verification of transparent, cubic crystals is sufficient. If there is white powder forming instead, it can be reasoned that this is not CD-MOF-1 forming but instead cyclodextrin precipitating out of solution.

For advanced courses:

For advanced courses where the instructor has access to an X-ray diffractometer and students would benefit from more in-depth materials characterization techniques, PXRD can be run using a few CD-MOF-1 samples. When students return materials to dispose of chemical waste, the instructor or TAs can run PXRD on a few CD-MOF-1 samples and send the data to the students. Student can then compare PXRD spectra of synthesized

MOF samples to predicted spectra generated by a computer model of the crystal structure. This alignment of experimental peaks with diagnostic simulated peaks, verifies synthesis of the predicted MOF structure. Instructor can explain crystal structures, crystallographic information files (CIFs) used to generate simulated spectra, as well as how X-ray diffraction works. Students can compare spectra to a 1:8 molar mixture of γ-CD: $C_7H_5KO_2$ (the MOF precursors) to ascertain that all precursor is no longer present, indicating purity of the MOF.

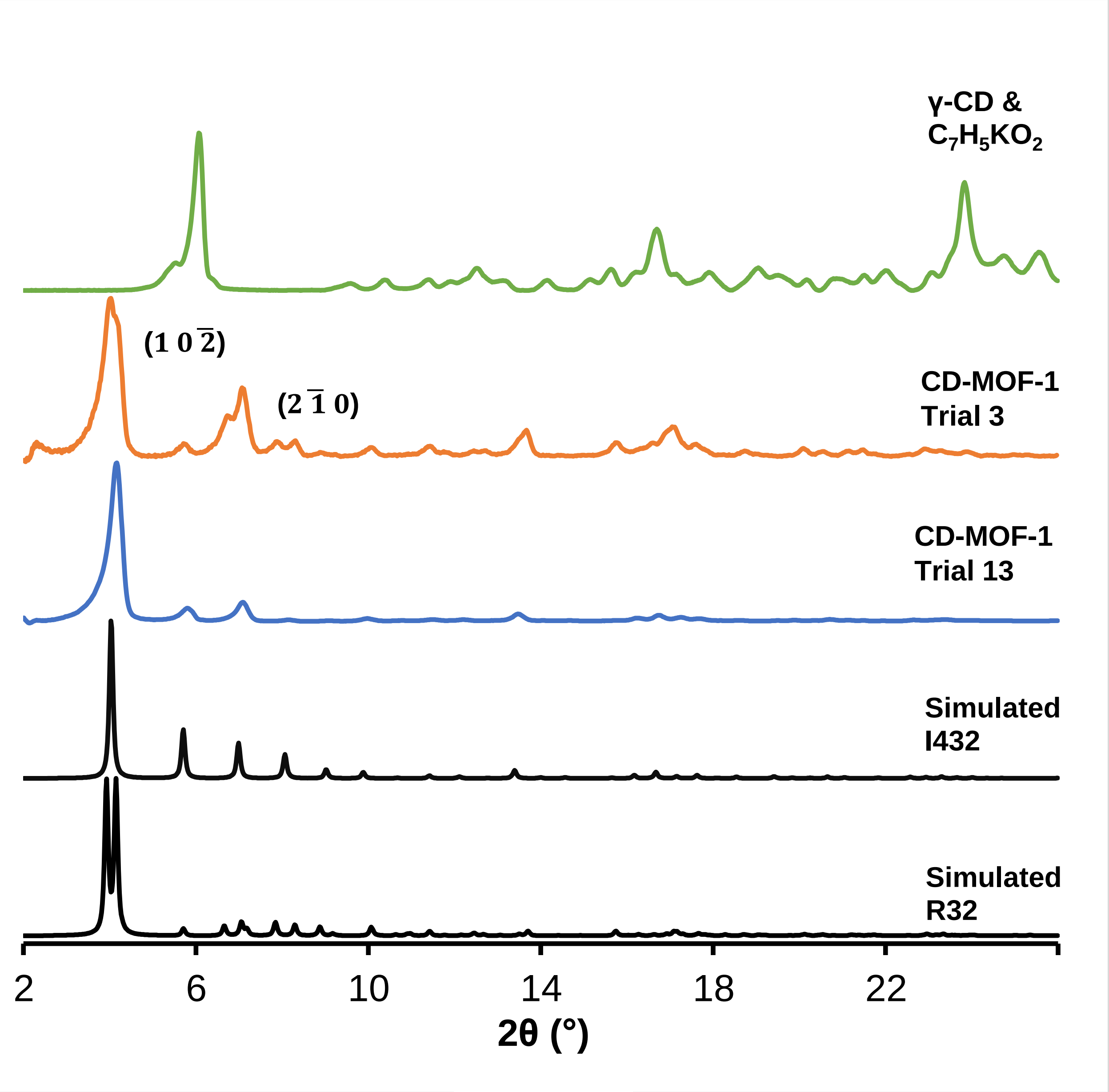

Figure S3: PXRD of CD-MOF-1 from trials 3 and 13. Experimental MOF peaks alight with peaks predicted from a computationally generated MOF structure using crystallographic information files (CIF) (773706.cif) (773711.cif). From the crystallographic data, the space group R32, the unit cell is trigonal.[1] The unit cell of the I432 space group is cubic.[1] Experimental PXRD of 1:8 molar ratio of γ-CD:$C_7H_5KO_2$ shows the change in peak position going from precursors to MOF crystal. Powder X-ray diffraction (PXRD) measurements were performed with a Rigaku sixth generation MiniFlex X-ray diffractometer with a 600 W (40 kV, 15 mA) CuKα (λ = 1.54 Å) radiation source.

V. **Student Handouts:**

Important Concepts

Spend time reading the following definitions and explore the links provided before you begin the experiment to better understand the concepts that we are about to explore!

- **Crystal:** solid composed of atoms, ions, or molecules arranged in a pattern that is periodic in three dimensions
- **Metal–Organic Framework (MOF):** class of coordination compounds consisting of metal ions or clusters coordinated to organic ligands to form two-, or three-dimensional structures in the solid state. The resulting structures are porous enabling them to serve as exceptional materials for gas storage.[3]

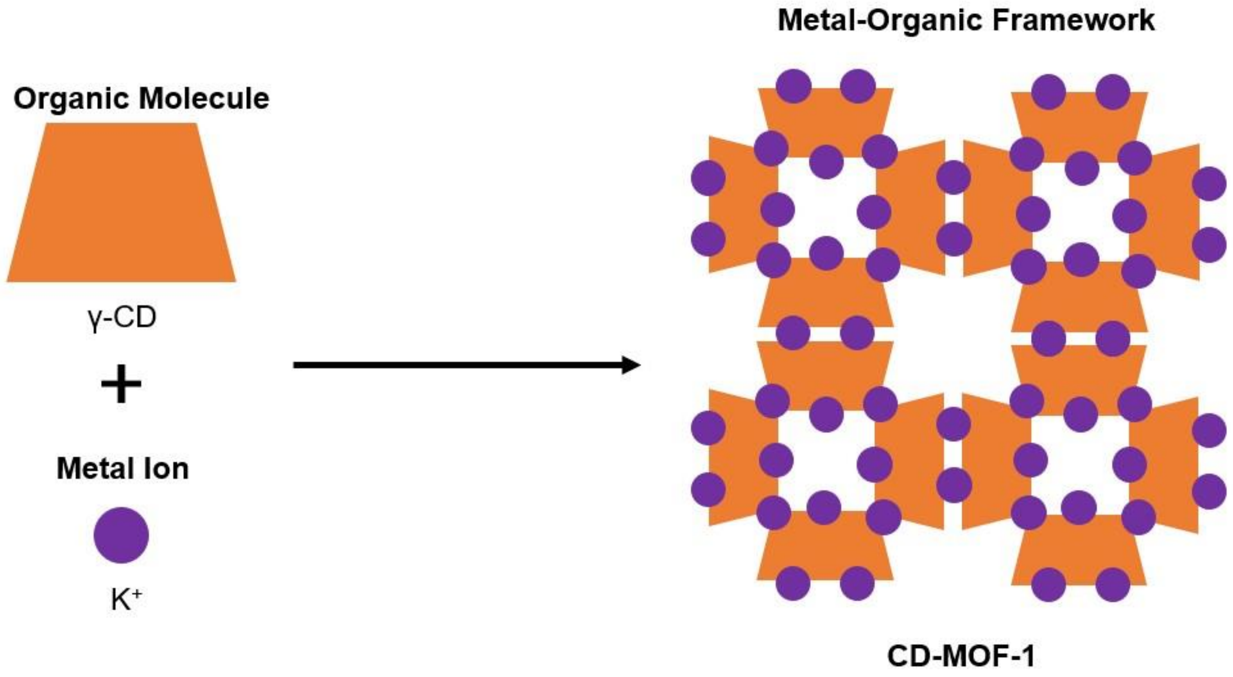

Pictorial representation of the formation of porous CD-MOF-1 from precursors gamma cyclodextrin and potassium ion.

- **Cyclodextrins**: a class of complex cyclic sugars that are products of the enzymatic decomposition of starch. Cyclodextrins can catalyze reactions between small molecules, which approach each other within the cylindrical body of the sugar. Cyclodextrins exist in three forms α, β, and γ, which correspond to six, seven, and eight glucopyranose units, respectively as seen below. The number of units determines the number of hydroxyl groups the molecule has. The hydroxyl groups serve as reactive functional groups to coordinate to inorganic compounds and form supramolecular frameworks. Therefore, the different number of hydroxyl groups in α-, β-, and γ-CD results in different structures. In the case of your experiment, γ-CD coordinates into cubes through bridges with potassium ions, which forms a porous MOF structure.

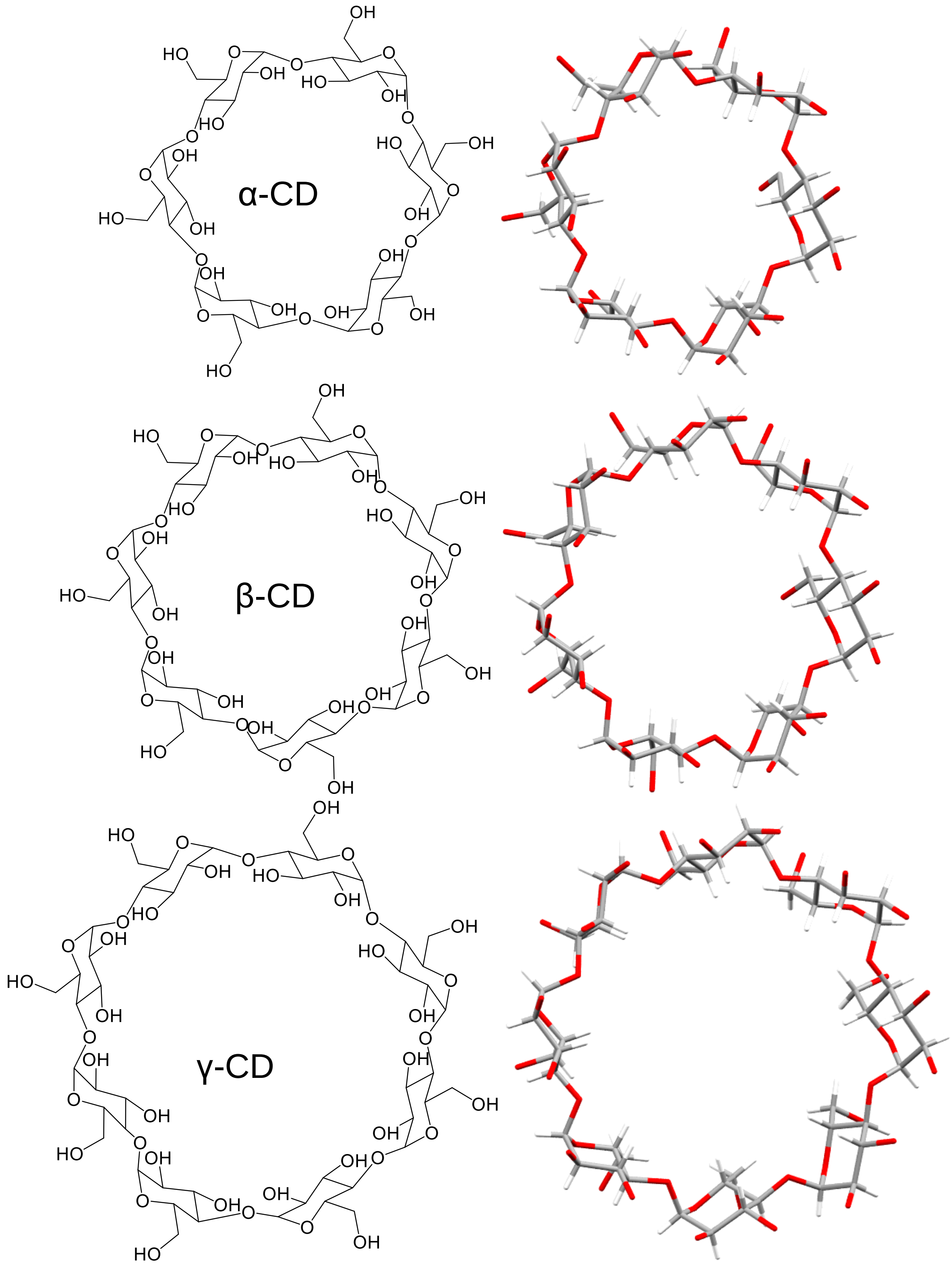

Chemdraw and stick models of α-, β-, and γ-cyclodextrin molecules.

- **Potassium:** chemical element of atomic number 19: in the alkali metal group
  - the potassium source used in this experiment is potassium benzoate ($C_7H_5KO_2$). The benzoate serves as a counterion for the positively charged potassium to form the salt. During MOF crystallization the K^+ ions coordinate the CD and the benzoate counterions are incorporated into the pores of the framework.

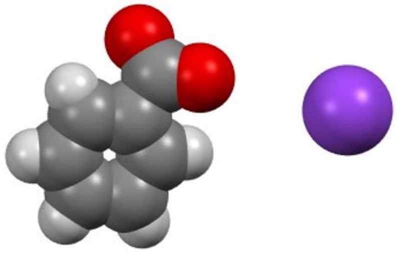

Space filling model of potassium cation and benzoate counter ion.

- **Coordination:** metal's coordination preference influences the size and shape of pores by dictating how many ligands can bind to the metal and in which orientation. The following citation details coordination frameworks and the geometrical constraints of the building blocks that determine framework shape and growth.[4]

- **Porous material:** a material containing pores (voids)

- **Green chemistry:** the design of chemical products and processes that reduce or eliminate the use or generation of hazardous substances. This is a relatively new field; the term "green chemistry" was coined in 1991.

- **12 Principles of Green chemistry**

The CD-MOF-1 was selected for this procedure because it possesses the ability to be synthesized from the bottom up from natural and benign precursors: γ-CD, a cyclic oligosaccharide, and potassium benzoate, a food-grade salt commonly used as a food additive or preservative.[1] The use of CD-MOF-1 and the experimental design adheres to the 8 out of the 12 principles of green chemistry. These benign precursors satisfy the following principles: 4. Designing Safer Chemicals, 7. Use of Renewable Feedstocks and 10. Design for Degradation. Through the use of ethanol in place of chlorinated solvents, the following green chemistry principle is satisfied: 5. Safer Solvent & Auxiliaries. The use of vapor diffusion at room temperature and ambient pressure the following principles are satisfied: 3. Less Hazardous Chemical Synthesis, and 12. Safer Chemistry for Accident Prevention. MOF activation satisfies 6. "Design for Energy Efficiency" as the crystals are dried under ambient conditions without a vacuum pump and/or vacuum. Finally, the color change of the activated crystals is concomitant to the reaction of baking soda and vinegar, which offers real-time assessment of CO_2 generation, which satisfies the 11. "Real-time analysis for Pollution Prevention" principle. [5]

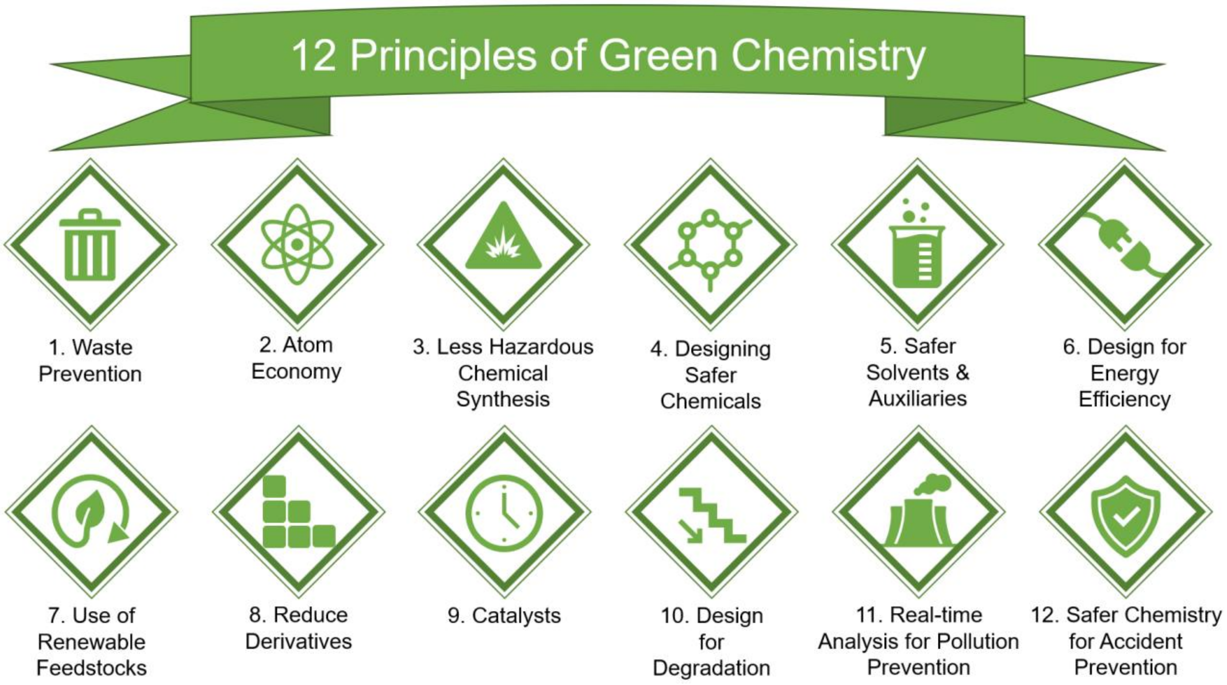

Links outlining green chemistry:

Introduction to green chemistry from McGill:[6]
https://www.youtube.com/watch?v=4PYxs8KBBHQ

Student Handouts Continued:

Procedure: Kit Provided Version: Materials and Supplies

i. **Chemicals**

1. 195 mg gamma cyclodextrin (γ-$C_{48}H_{80}O_{40}$) in a 20 mL glass vial labeled **"Gamma Cyclodextrin (γ-CD)"**
2. 192.4 mg potassium benzoate ($C_7H_5KO_2$) in a 20 mL plastic vial labeled **"Potassium Benzoate"**
3. 4 mL 95% ethanol (EtOH) in a 20 mL plastic vial labelled **"4 mL EtOH"**
4. 1 mL deionized water (DI H_2O) in a 20 mL plastic vial labelled **"1 mL DI H_2O"**
5. 7.7 mg of methyl red sodium salt labelled **"7.7 mg methyl red"** and 20 mL 95% EtOH labelled **"20 mL EtOH"** to make 1.32 mM stock solution
6. 0.4 g sodium bicarbonate ($NaHCO_3$) in a 20 mL plastic vial labeled **"0.4 g $NaHCO_3$"**
7. 5 mL white vinegar (5% acetic acid (5% CH_3COOH)) in a 20 mL plastic vial labeled **"5 mL 5% CH_3COOH"**

ii. **Supplies**

1. 3 mL syringe (one)
2. Cotton ball (one)
3. Forceps
4. 1 (one) 20 mL scintillation vial
5. 1 (one) 1.8-dram vials
6. 3 mL disposable plastic pipettes (five)
7. Safety glasses
8. Nitrile Gloves

Optimized Procedure for CD-MOF-1 Crystal Growth by Vapor Diffusion: Kit Provided Procedure

The steps below outline the synthetic steps for the optimized crystallization of CD-MOF-1 using the prepared kit.

Step 1:

Put on proper PPE and collect chemicals #1−4 and supplies #1−5.

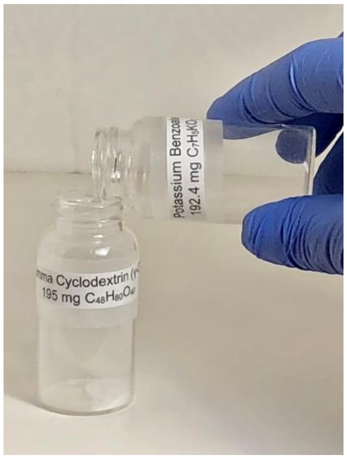

Step 2:

Transfer contents from vial labelled **“Potassium Benzoate”** (192.4 mg) to the **“Gamma Cyclodextrin (γ-CD)”** (195 mg) vial to combine the potassium benzoate and gamma cyclodextrin precursors. Note: Static may make transfer difficult. Try putting vials mouth to mouth and tapping **“Potassium Benzoate”** vial to transfer.

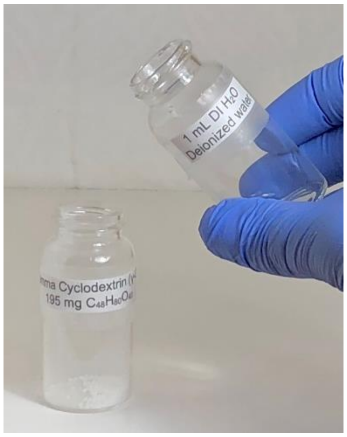

Step 3:

Pour deionized water from vial labelled **“1 mL DI H_2O”** to the **“Gamma Cyclodextrin (γ-CD)”** vial. Cap the vial and gently swirl to facilitate the dissolving of the precursors. Note: the dissolving takes a few minutes. You can leave the solution for a few minutes and then continue stirring the vial.

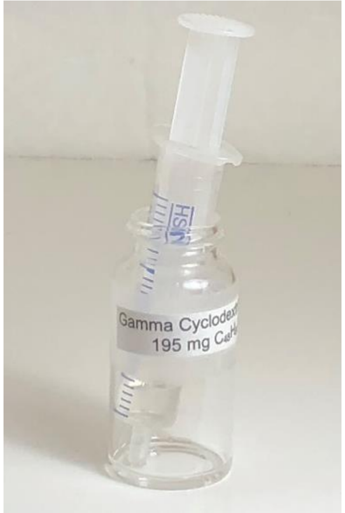

Step 4:

Draw up 1 mL of the solution with the dissolved MOF reagents using the syringe. Try to avoid excessive bubbles in syringe.

Step 5:
Form a filter from a very small piece of a cotton ball by rolling it between the pointer finger and thumb.

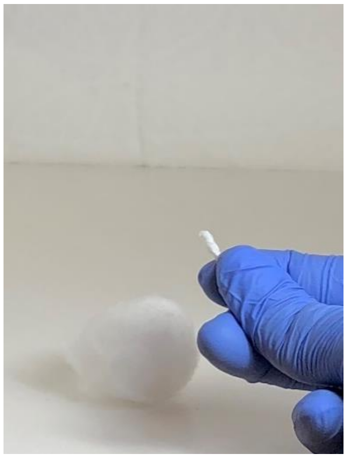

Step 6:
To enable filtering of the solution before its transfer to the 1.8 mL vial, draw up the precursor solution using the syringe and then invert the syringe so the nozzle is solution free. Place the small piece of a cotton ball in the nozzle to serve as a filter.

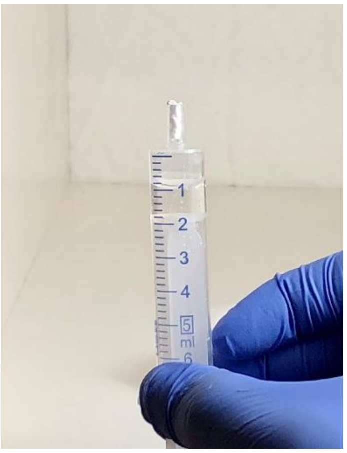

Step 7:
Filter the solution in the syringe into the unlabeled and empty 1.8 mL dram vial. (Note: press plunger gently so that the cotton filter does not dislodge.) By filtering we are removing any undissolved cyclodextrin or potassium benzoate that will interfere with MOF crystal growth.

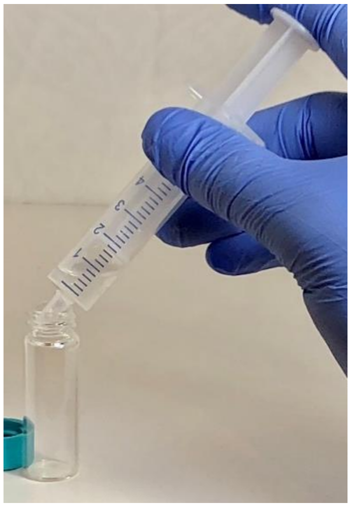

Step 8:

Add 4 mL of 95% ethanol from the vial labelled “**4 mL EtOH”** to the empty, unlabeled 20 mL glass vial.

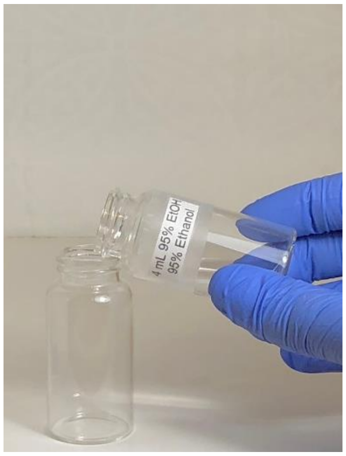

Step 9:

Using tweezers (small tongs work as well) carefully lower the unlabeled, uncapped 1.8 mL vial with the CD/potassium benzoate solution into the 20 mL scintillation vial so that the meniscus of the ethanol is above the meniscus of the inner vial. (Note: Be careful so that the inner vial does not tip or get ethanol in it.)

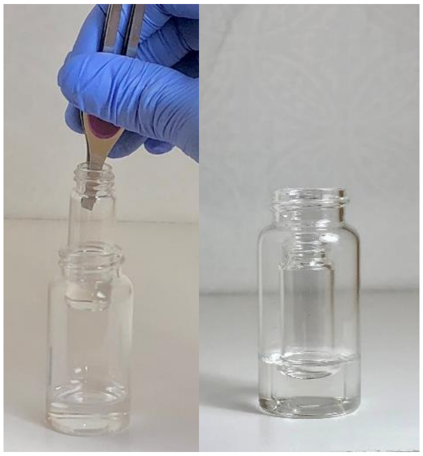

Step 10:

Cap the outer vial tightly. (Note: Move set up carefully so that the inner vial does not tip).

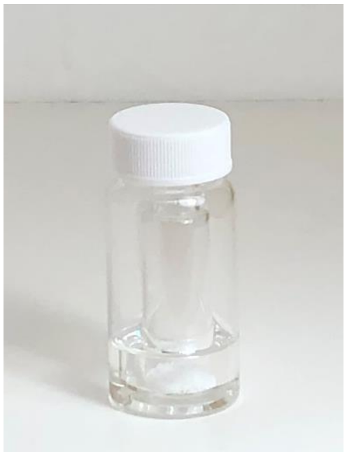

Step 11:

Leave experimental set up undisturbed in a dark location for 3−7 days for vapor diffusion to allow for proper crystal growth. Observe the vial over the course of the 3−7 days to monitor crystal growth. (Note: white powder is the cyclodextrin precipitating of solution and is not the desired MOF. Cubic, clear larger crystals (1−3 mm) are the desired CD-MOF-1.

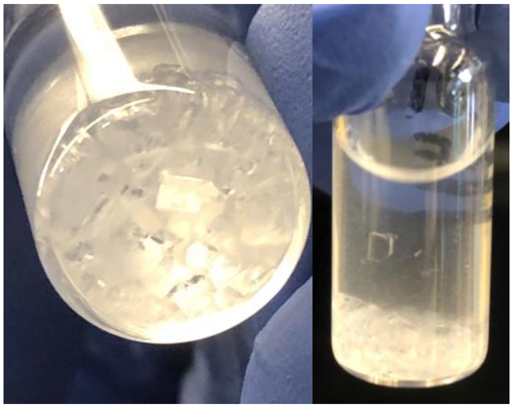

Optimized Procedure for CD-MOF-1 Activation and Methyl Red Incorporation: Kit Provided Procedure

The steps below outline the steps for the optimized activation of CD-MOF-1 and incorporation of the methyl red indicator into the framework pores using the prepared kit.

Step 1:

Put on proper PPE and collect chemical #5 and supply #6.

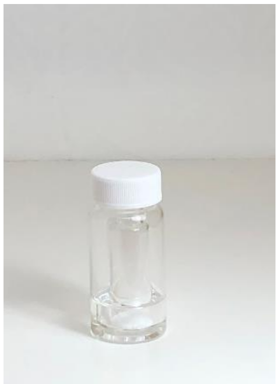

Step 2:

Remove the inner vial containing crystals from the larger vial and dry outside of vial with a paper towel. Pipet out the solvent (now DI water and 95% ethanol due to vapor diffusion) in the inner vial surrounding the crystals and dispose of it properly.

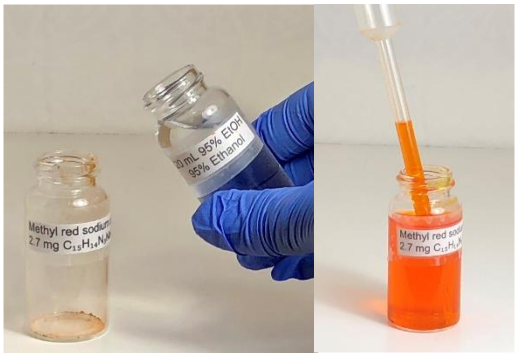

Step 3:

Make methyl red stock solution by adding 20 mL 95% ethanol to 7.7 mg methyl red sodium salt. Use pipette to ensure proper mixing to dissolve solid.

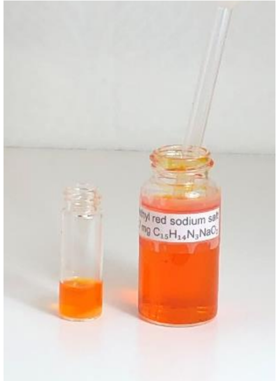

Step 4:

From the methyl red stock solution, pipette a small amount of methyl red solution into the small vial with crystals using a 3 mL plastic disposable pipette so that all the crystals are completely submerged.

Step 5:

Cap vial and leave the crystals to soak for 1−24 hours to allow for proper diffusion. Over the course of the diffusion, it is possible to see the crystals change from slightly transparent to yellow.

Step 6:

After 1−24 hours, pipette the methyl red indicator solution out and dispose of it properly. It helps to tip the vial while pipetting to avoid disturbing crystals. The crystals should look light yellow. (Note: If the crystals did not look yellow, the time for solvent exchange was increased by leaving the crystals submerged in the methyl red solution for longer.)

Step 7:

Steps 4–6 were repeated.

Step 8:

To activate the crystals (remove any extra solvent trapped in the pores) pipette a small amount of 95% ethanol into the small vial and leave for 1−24 hours. After 1−24 hours, pipette out and dispose of the ethanol. This step is to complete the activation of the MOF and evacuate the rest of the water from the pores of the MOF.

Step 9:

Lightly cap the small vial of MOF crystals and leave for 2 days to dry. The final activated crystals look yellow. If left uncapped for a long time, the edges of the crystals will darken to a deeper orange, which we want to avoid.

Note: Drying can be accelerated if humidity is a concern using a vacuum system, or desiccator. If there is no access to this equipment, the drying time can be extended until completed.

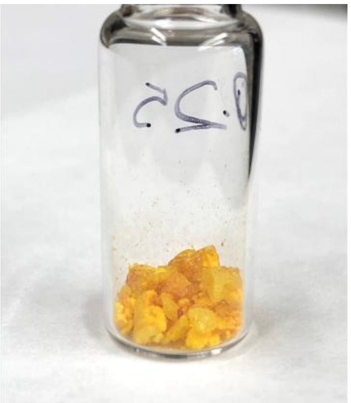

Optimized Procedure for Colorimetric Analysis of CO_2 Uptake by CD-MOF-1: Kit Provided Procedure

The steps below outline the procedure for the optimized exposure of CD-MOF-1@Methyl Red to CO_2 using the prepared kit. This qualitative observation of the color change of the indicator shows CO_2 incorporation.

Step 1:

Put on proper PPE and collect chemicals #6−7 and supplies #6.

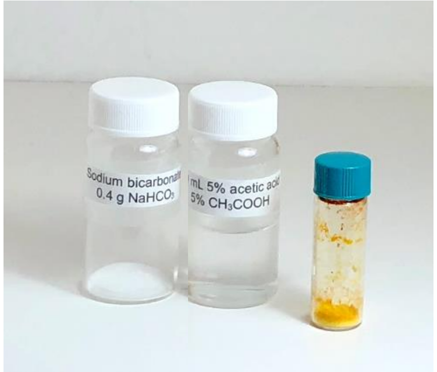

Step 2:

Take note of the color of the CD-MOF-1 crystals before CO_2 exposure.

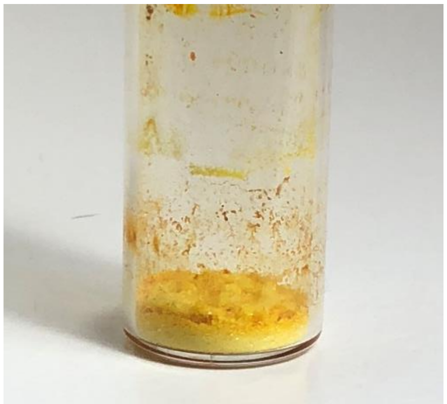

Step 3:

Uncap small vial with activated CD-MOF-1@methyl red crystals. Add the vial to the larger vial labeled "**Sodium bicarbonate**" using tweezers or tongs.

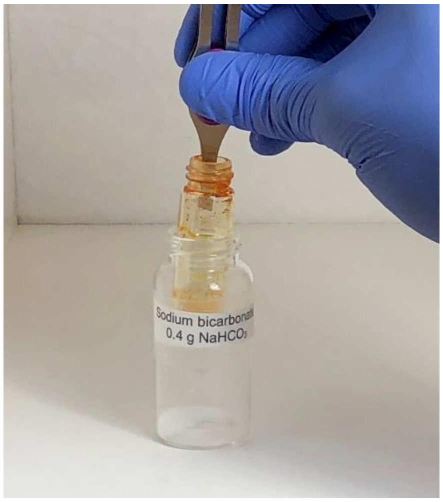

Balanced Reaction for CO_2 production:

$$NaHCO_3 + CH_3COOH \rightarrow NaC_2H_3O_2 + CO_2 + H_2O$$

Step 4:

Using a pipette, add 5 mL of "**5% acetic acid**" to the outer vial labelled "**Sodium bicarbonate**". This will begin the vinegar-baking soda reaction. Add the vinegar in two aliquots (portions) so that the reaction does not bubble into the small vial. Add the vinegar quickly so that not all the CO_2 escapes before you can add the cap on. However, be careful not to drop vinegar into the smaller inner vial as this will artificially change the color of the indicator (since acid would be in direct contact with the indicator).

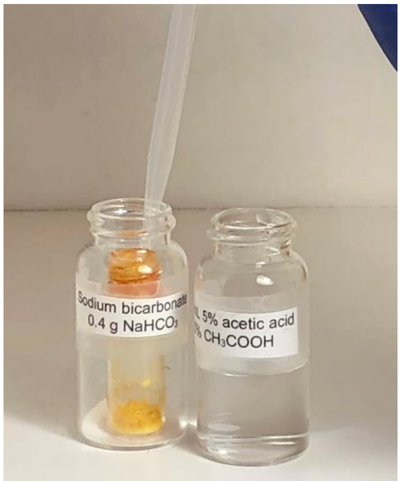

Step 5:

Lightly cap the vial once all the vinegar has been added. Observe the color change as the vinegar-baking soda reaction progresses and produces CO_2 that diffuses into the small vial.

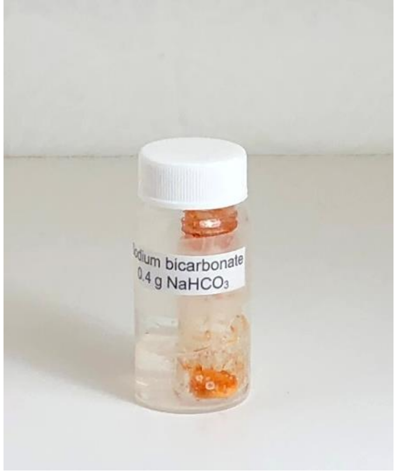

Step 6:

Once the vinegar-baking soda reaction ceases (no more gas and bubbles evolving) remove the inner vial with the CD-MOF-1@methyl red crystals to better observe the color change.

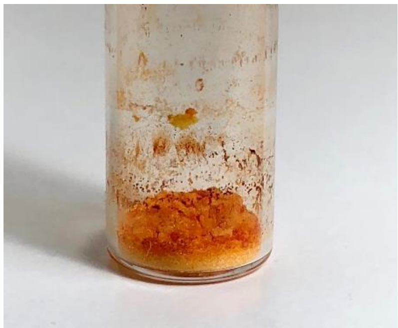

Step 7:

Leave the CD-MOF-1@methyl red crystals uncapped for about 20 minutes to enable CO_2 desorption and observe the color change back to yellow.

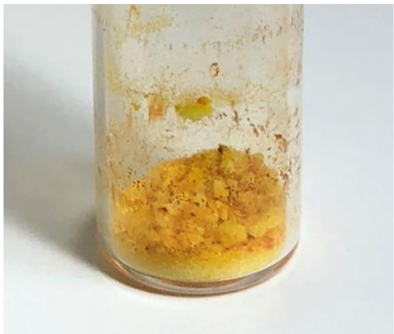

Procedure: Independent Experiment: Materials and Supplies

i. Chemicals from Manufacturers

1. Food grade gamma cyclodextrin (γ-$C_{48}H_{80}O_{40}$) (TGCD-F) was sourced from CTD, Inc. (Alachua, FL)
2. Potassium benzoate ($C_7H_5KO_2$) (EG-C7H5KO2-4) was sourced from Eisen-Golden Laboratories and purchased on ebay.com for $8.99
3. 95% ethanol (UN1170) was sourced from Home Science Tools (Billings, MT) ($5.35/ 30mL)
4. Deionized water (4 mL) in vial labelled "**DI H_2O**"
5. Methyl red, sodium salt ($C_{15}H_{14}N_3NaO_2$) (845-10-3) was sourced from Fisher Science Education ($11.25/10g)

ii. Chemicals Available for Purchase in Grocery Stores

1. Sodium bicarbonate (Baking soda ($NaHCO_3$))
2. 15 mL white vinegar (5% acetic acid (5% CH_3COOH))

iii. Supplies from Manufacturers

1. Scale (AMIR Digital Kitchen Scale, 500 g)
2. 3 mL NORM-JECT syringe (one)
3. Forceps
4. 20 mL scintillation vial with cap (four)
5. 1.8-dram vial with cap (one)
6. Pipettes (five) (plastic)
7. Safety glasses
8. Nitrile gloves

iv. Supplies Available for Purchase in Stores

1. Cotton ball (one)

Optimized Procedure for CD-MOF-1 Crystal Growth by Vapor Diffusion: Independent Procedure

The steps below outline the synthetic steps for the optimized crystallization of CD-MOF-1 using the independent procedure.

Step 1:

Put on proper PPE and collect chemicals from manufacturers #1−4, supplies from manufacturers #1−5, and supplies from stores #1.

Step 2:

Measure out 390 mg of Gamma cyclodextrin (γ-CD) and 384.8 mg potassium benzoate ($C_7H_5KO_2$) using a small digital scale. Transfer the reagents to 2 mL of DI water. Cap the vial and gently swirl to facilitate the dissolving of the precursors. Note: the dissolving takes a few minutes. You can leave the solution for a few minutes and then continue stirring the vial.

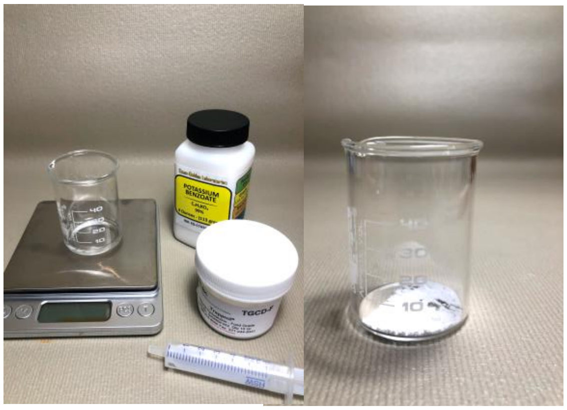

Step 3:

Draw up 1 mL of the solution with the dissolved MOF reagents using the syringe. Try to avoid excessive bubbles in syringe.

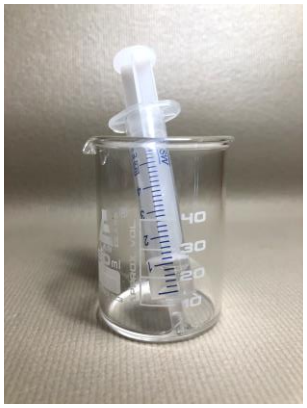

Step 4:

Form a filter from a very small piece of a cotton ball by rolling it between the pointer finger and thumb.

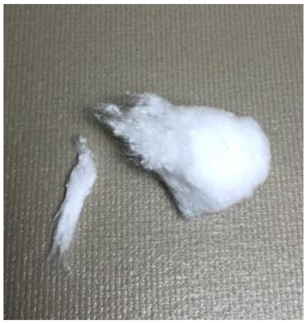

Step 5:

Invert the syringe and pull the plunger slightly to draw up about 0.2−0.4 mL of air to make room for the cotton filter. Put cotton into nozzle to serve as a filter. Make sure it fits snuggly so that it does not dislodge easily in step **6**.

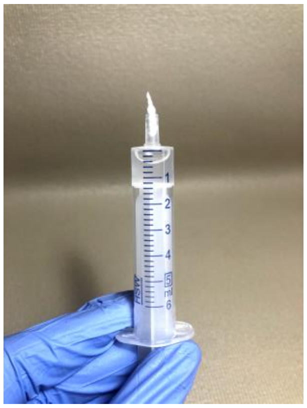

Step 6:

Filter the solution in the syringe into the unlabeled and empty 1.8 mL dram vial. (Note: plunger was pressed gently so that the cotton filter does not dislodge.) By filtering we are removing any undissolved cyclodextrin or potassium benzoate that will interfere with MOF crystal growth.

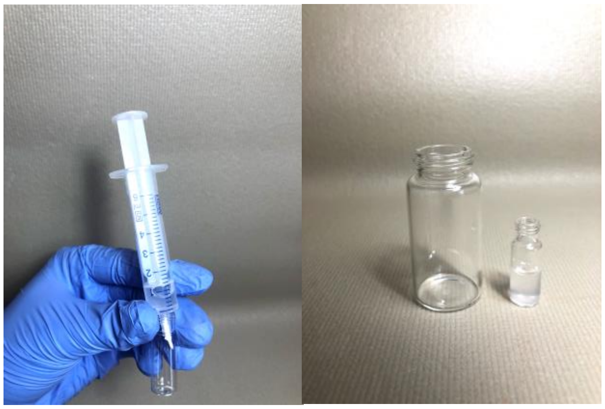

Step 7:

Fill a 20 mL scintillation vial with about 4 mL 95% ethanol. Replace 95% ethanol with 91% isopropanol if that is what you have available.

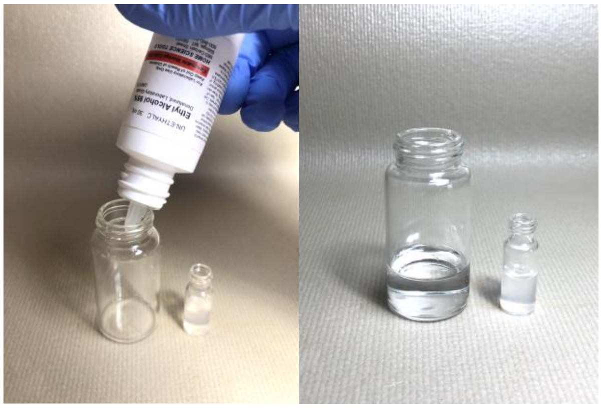

Step 8:

Using tweezers (small tongs would work as well) transfer the 1.8 mL vial with MOF reagent solution carefully into the 20 mL scintillation vial.

The meniscus of the ethanol should be above the meniscus of the inner vial. (Note: Be careful so that the inner vial does not tip or get ethanol in it.)

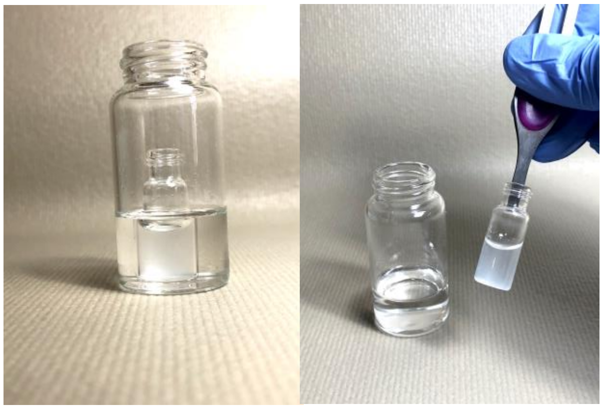

Step 9:

Cap the outer vial tightly. (Note: Move set up carefully so that the inner vial does not tip).

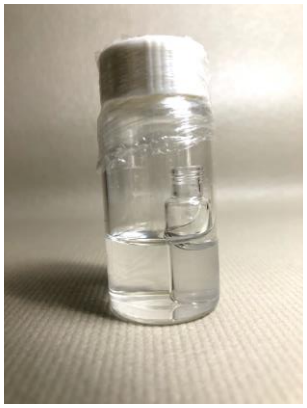

Step 10:

Leave experimental set up undisturbed in a dark location for 3−7 days for vapor diffusion to allow for proper crystal growth. Observe the vial over the course of the 3−7 days to monitor crystal growth. (Note: white powder is the cyclodextrin precipitating of solution and is not the desired MOF. Cubic, clear larger crystals (1−3 mm) are the desired CD-MOF-1.

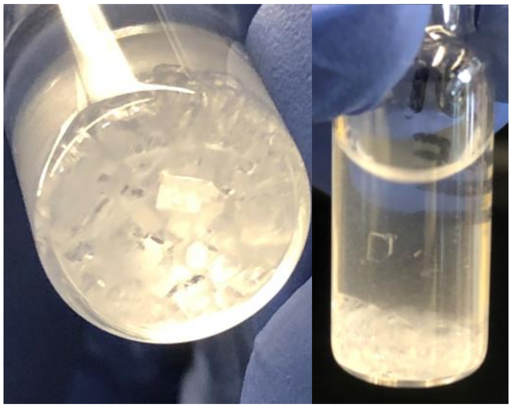

Optimized Procedure for CD-MOF-1 Activation and Methyl Red Incorporation: Independent Procedure

Optimized activation of CD-MOF-1 with methyl red indicator using the independent experiment procedure. The methyl red solution was made with ethanol as a more safe and environmentally benign alternative to the use of DCM, which was previously published.

Step 1:

Put on proper PPE and collect chemicals #3 & #5 and supplies #6.

Step 2:

Remove the inner vial containing crystals from the larger vial and dry it. Pipette out the solvent (now DI water and alcohol due to vapor diffusion) in the inner vial surrounding the crystals.

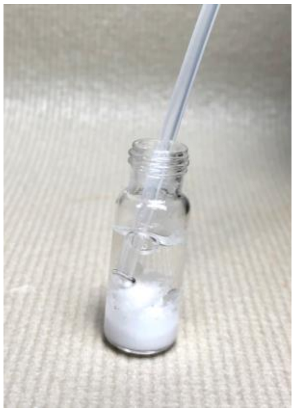

Step 3:

Make methyl red stock solution by adding 20 mL 95% ethanol to 7.7 mg methyl red sodium salt. Use pipette to ensure proper mixing to dissolve solid.

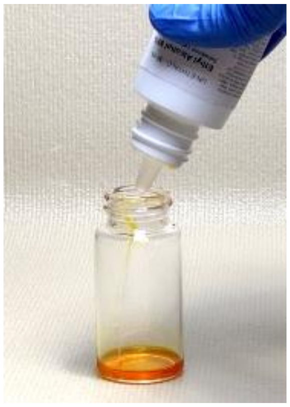

Step 4:

From the methyl red stock solution, pipette a small amount of methyl red solution into the small vial with crystals using a 3 mL plastic disposable pipette so that all the crystals are completely submerged.

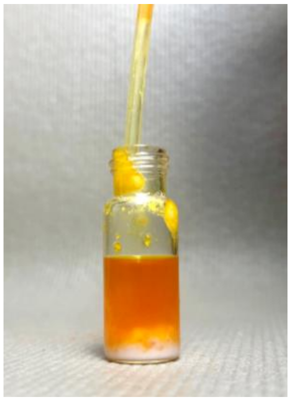

Step 5:

Cap vial and leave the crystals to soak for 1−24 hours to allow for proper diffusion. Over the course of the diffusion, it is possible to see the crystals change from slightly transparent to yellow.

Step 6:

After 1−24 hours, pipette the methyl red indicator solution out and dispose of it properly. It helps to tip vial while pipetting to avoid disturbing crystals. The crystals should look light yellow. (Note: If the crystals did not look yellow, the time for solvent exchange was increased by leaving the crystals submerged in the methyl red solution for longer.)

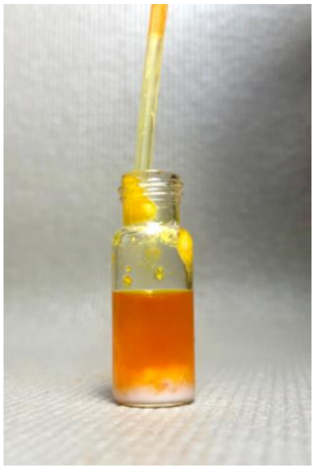

Step 7:

Steps 4–6 were repeated.

Step 8:

To activate the crystals (remove any extra solvent trapped in the pores) pipette a small amount of 95% ethanol into the small vial and leave for 1−24 hours. After 1−24 hours, pipette out and dispose of the ethanol. This step is to complete the activation of the MOF and evacuate the rest of the water from the pores of the MOF.

Step 9:

Lightly cap the small vial of MOF crystals and leave for 2 days to dry. The final activated crystals look yellow. If left uncapped for a long time, the edges of the crystals will darken to a deeper orange, which we want to avoid.

Note: Drying can be accelerated if humidity is a concern using a vacuum system, or desiccator. If there is no access to this equipment, the drying time can be extended until completed.

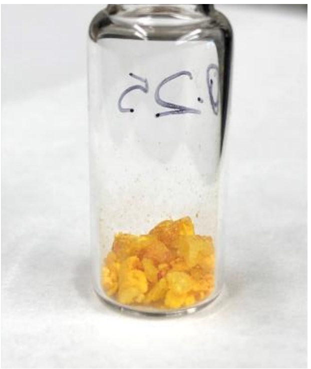

Optimized Procedure for Colorimetric Analysis of CO_2 Uptake by CD-MOF-1: Independent Procedure

Step 1:

Put on proper PPE and collect chemicals from store #1−2 and supplies #4 & #6.

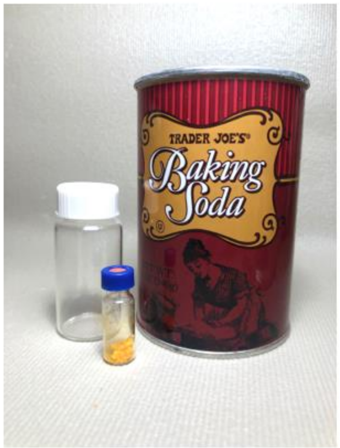

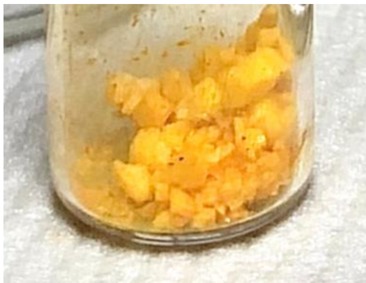

CD-MOF-1 before CO_2 exposure

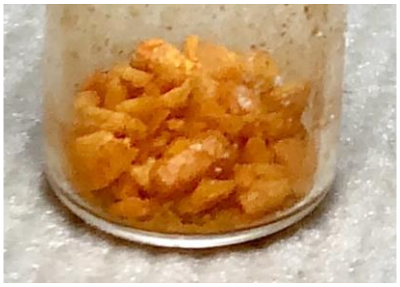

CD-MOF-1 after CO_2 exposure

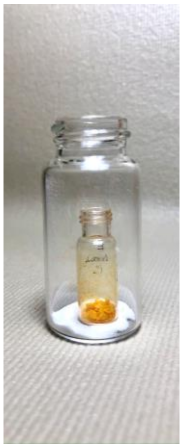

1. Add 0.4 g baking soda to scintillation vial and use tweezers to add small vial with MOF in.

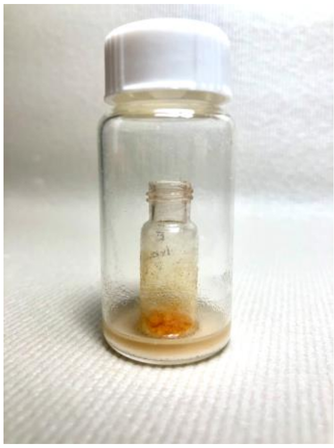

2. Use a pipet to add 5 mL of vinegar in two aliquots to the outer vial and quickly cap. Add the vinegar quickly so that not all the CO_2 escapes before you can add the cap on. However, be careful not to drop vinegar into the smaller inner vial as this will artificially change the color of the indicator (since acid would be in direct contact with the indicator).

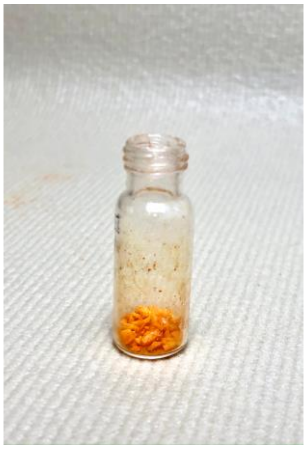

3. Cap the vial and allow 5 minutes for enough CO_2 to evolve and diffuse into MOF pores

4. Remove inner vial to observe color change.

Balanced Reaction for CO_2 production:

$$NaHCO_3 + CH_3COOH \rightarrow NaC_2H_3O_2 + CO_2 + H_2O$$

Step 5:

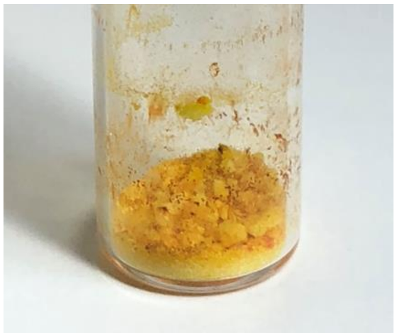

Leave the CD-MOF-1@methyl red crystals uncapped for about 20 minutes to enable CO_2 desorption and observe the color change back to yellow.

VI. Safety Instructions

Always wear safety glasses, gloves, and protective clothing (long sleeves, long pants, and shoes) when conducting the experiment.

Chemical Included:

-Potassium Benzoate

-Cyclodextrin

-Methyl Red sodium salt

-Ethanol

According to the European Standard (EN 71-4) Chemical Substances in Experimental Sets, "Apart from its presence in tincture of iodine, denatured alcohol (ethanol) shall not be supplied in a *chemistry set*. However, where experiments contained in the instructions of a *chemistry set* require it, the use of denatured alcohol may be suggested in the instructions."

According to the European Standard (EN 71-4) Chemical Substances in Experimental Sets, Methyl Red Sodium Salt indicator can be included in chemistry sets as it does "not react with the substances and mixtures of the set and if they do not fulfil the criteria of any of the following hazard classes:

- 'acute toxicity' (hazard class 3.1),
- 'skin corrosion/irritation' (hazard class 3.2),
- 'serious eye damage/eye irritation' (hazard class 3.3),
- 'respiratory or skin sensitisation' (hazard class 3.4),
- 'germ cell mutagenicity' (hazard class 3.5),
- 'carcinogenicity' (hazard class 3.6),
- 'reproductive toxicity' (hazard class 3.7),
- 'specific target organ toxicity — single exposure' (hazard class 3.8),
- 'specific target organ toxicity — repeated exposure' (hazard class 3.9),
- 'aspiration hazard' (hazard class 3.10)"

The hazards were found using Methyl Red sodium salt SDS. All are not classified.[7]

Disposal Methods:

-Solutions of ethanol (any alcohol) over 24% may not be disposed of down the drain regardless of quantity or dilution.[8]

-Methyl Red sodium salt SDS
Cannot be disposed of down the sink.[7]

All chemical waste generated should be disposed of in according to proper procedures required by county, state, and country in which the experiment is run.

VII. Highlights of Results from High School Students

The figures below are photo images of notebooks used by high school students to document the experiment. There is also an excerpt from a student's notebook detailing what they learned from the experiment and its impact on their understanding of chemical concepts.

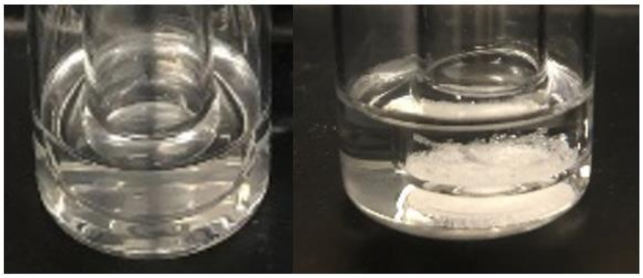

Figure S4: CD-MOF-1 vapor diffusion set up (left) and crystal growth after three days (right) performed by an AP chemistry high school student from Lebanon high school.

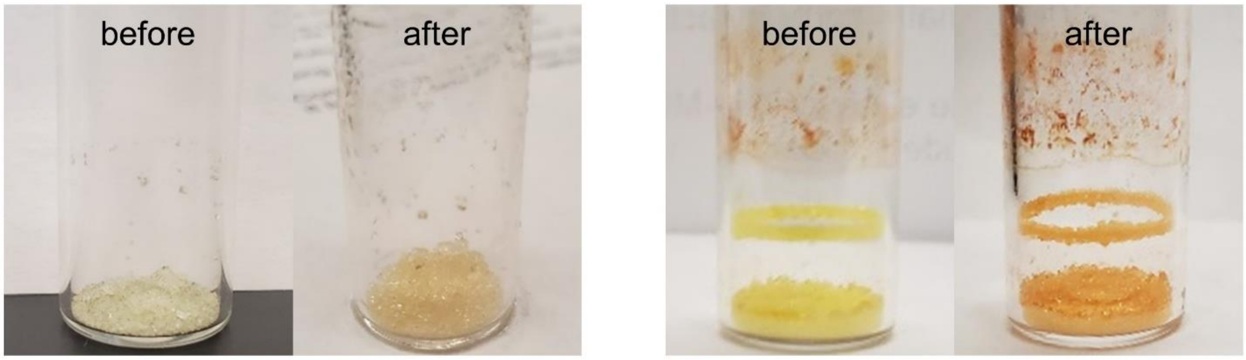

Figure S5: Images collected by two high school students from Lebanon High School depicting qualitative data collected from two CO_2 uptake experiments. The color of activated CD-MOF-1@methyl red crystals is documented before and after exposure to CO_2. The change in color from yellow to orange indicates CO_2 uptake by the MOF.

REFLECTION

"This experiment has allowed me to make connections between the concepts I have learned and their real-world applications. In the process of understanding this procedure, I now understand vapor diffusion and how complex structures like MOFs and crystals can be created with the gradual incorporation of an insoluble solution to a soluble solution. In this experiment, the solvent is less hospitable to solubility, so when the ethanol is gradually incorporated into the solution, the process of crystallization occurs as the MOFs become insoluble in solution. I also learned that reticular synthesis is the building up of complex network structures like MOFs and other crystals, and through the process of vapor diffusion, extremely complex structures can be formed if the process happens slowly enough. Host-guest chemistry was also a relatively new concept to me until this experiment. Due to their structure, MOFs have the capacity to "trap" various different gases and molecules. They are made up of a network of organic material held together by metal particles. MOFs have a number of interstices in which large amounts of particles can be contained in a relatively small volume."

VIII. Highlights of Results from Undergraduate Reports

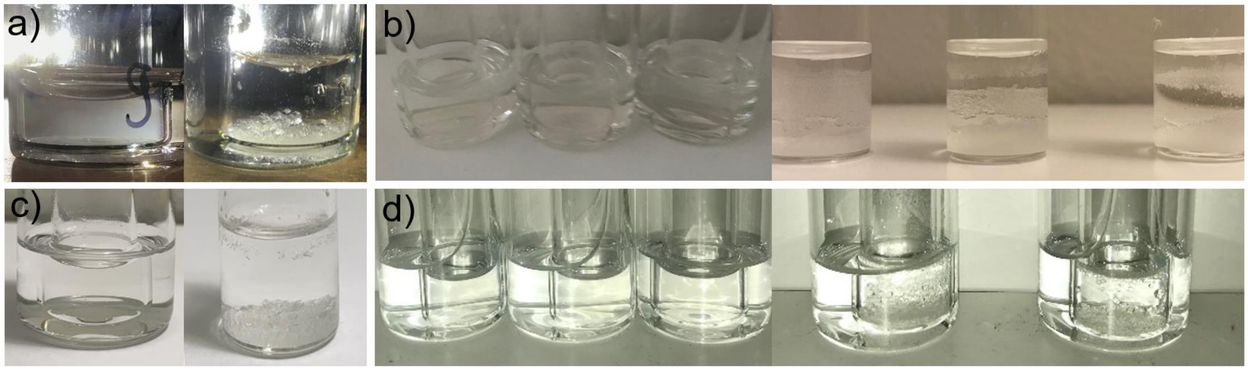

Figure S6: CD-MOF-1 vapor diffusion set up (left) and crystal growth after three days (right) performed by four Dartmouth undergraduate students (A−D). Students B and D took images of the experiment in triplicate.

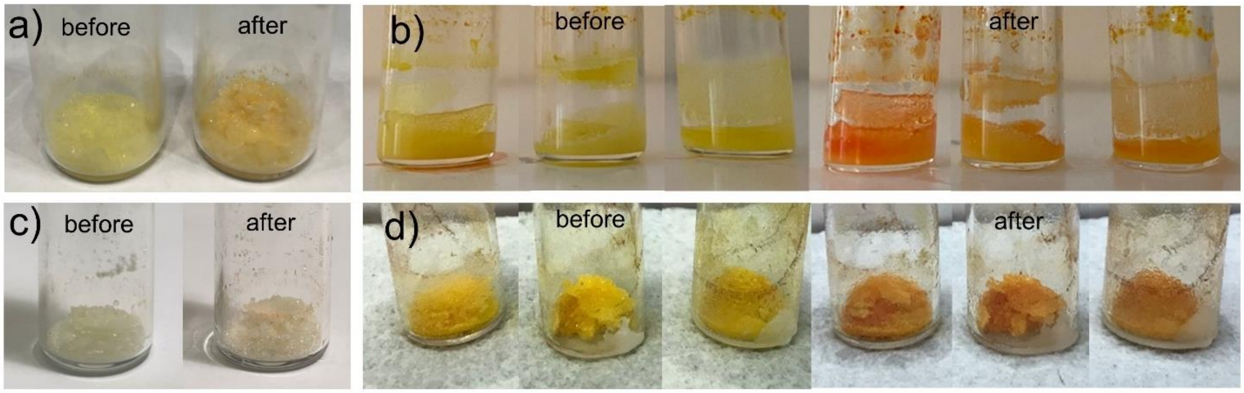

Figure S7: Adsorption of CO_2 by CD-MOF-1 crystals activated with methyl red resulting in the visible color change. Crystals change from yellow to orange due to CO_2 exposure. Images provided by four Dartmouth undergraduate students (A−D).

IX. References:

(1) Smaldone, R. A.; Forgan, R. S.; Furukawa, H.; Gassensmith, J. J.; Slawin, A. M.; Yaghi, O. M.; Stoddart, J. F. Metal-organic frameworks from edible natural products. *Angew Chem Int Ed Engl* **2010**, *49* (46), 8630-8634.
(2) O'Keeffe, M. *Crystal Structures: Patters and Symmetry*; Dover, 1996.
(3) *Metal-Organic Frameworks*. novoMOF, 2021. https://novomof.com/metal-organic-frameworks/?hsCtaTracking=ec8597ef-7650-4f00-9975-26a88ba24130%7C8d8099f6-9030-4abd-8c17-bc155c09fe0a.
(4) *Coordination Frameworks*. LibreTexts, 2022. https://chem.libretexts.org/Bookshelves/Inorganic_Chemistry/Map%3A_Inorganic_Chemistry_(Miessler_Fischer_Tarr)/09%3A_Coordination_Chemistry_I_-_Structure_and_Isomers/9.06%3A_Coordination_Frameworks.
(5) Hems, R. *Green Chemistry 101*. Let's Talk Science, 2019. https://letstalkscience.ca/educational-resources/stem-in-context/green-chemistry-101.

(6) University, M. Green Chemistry. https://www.youtube.com/watch?v=4PYxs8KBBHQ.
(7) *Methyl Red, Sodium Salt, ACS*; 58; LabChem, Zelienople, PA, 2014.
(8) *Industry Protocols for Proper Waste Disposal of Ethanol*. Weebly, https://ethanolimpacts.weebly.com/proper-waste-disposal.html#:~:text=In%20a%20laboratory%20and%20industrial,in%20the%20appropriate%20waste%20container.
