# Supplementary material for: CD-MOF-1 for CO2 Uptake: Remote and Hybrid Green Chemistry Synthesis of a Framework Material with Environmentally Conscious Applications
Source: J Chem Educ. 2023 Feb 13;100(3):1289–95. doi: 10.1021/acs.jchemed.2c00922 (PMC10018730; doi:10.1021/acs.jchemed.2c00922)
Supplement: Supplementary file 2 — ed2c00922_si_002.pdf [file ed2c00922_si_002.pdf]

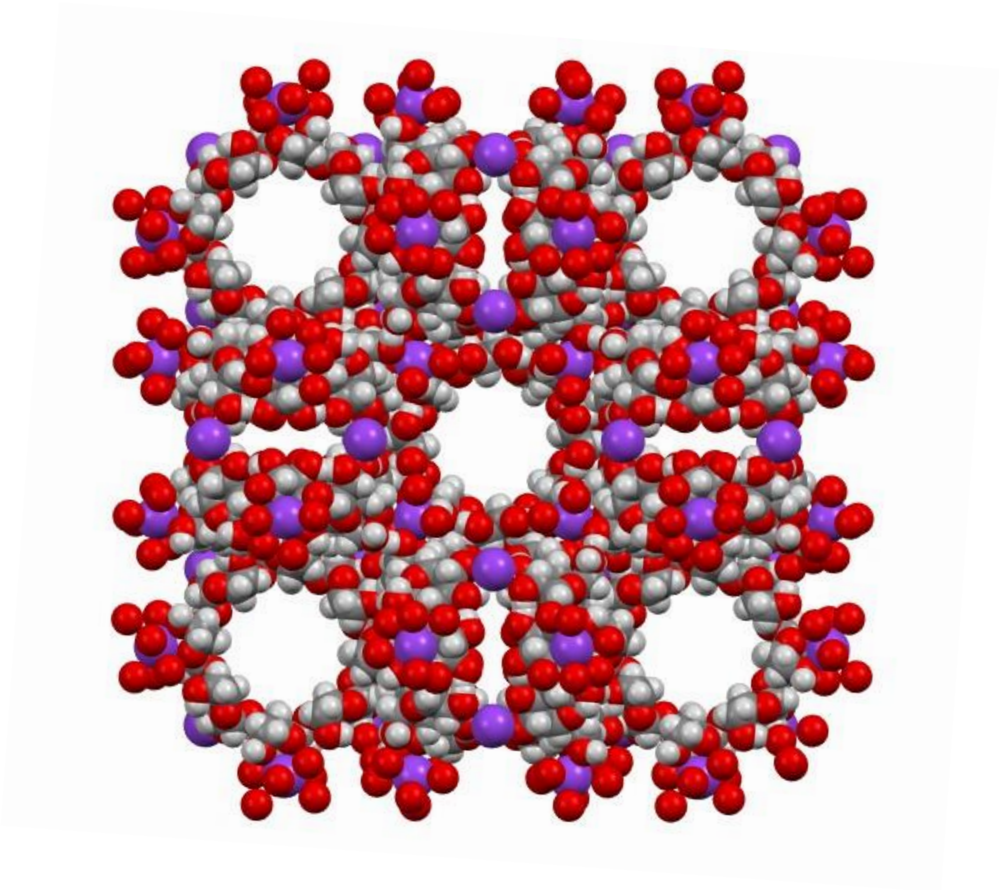

CD-MOF-1 for CO_2 Uptake: Remote and Hybrid Green Chemistry Synthesis of a Framework Material with Environmentally Conscious Applications

Background

- Greenhouse gas emissions pose a detrimental threat to the environment and human society
- Remedy: **sequestration** of CO_2 by porous green materials
- Green material of interest: Metal−organic frameworks (MOFs)
    - High surface area for efficient gas uptake
    - Synthesized using inexpensive, environmentally benign precursors
    - Thermally stable
    - Abide by many principles of green chemistry

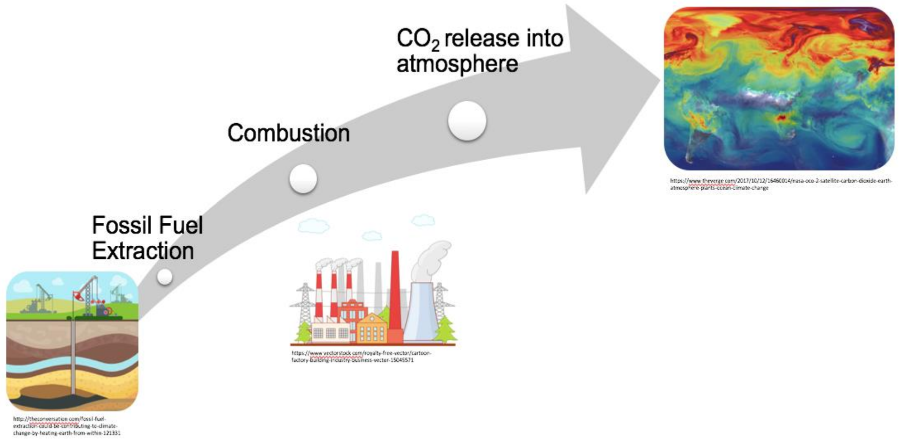

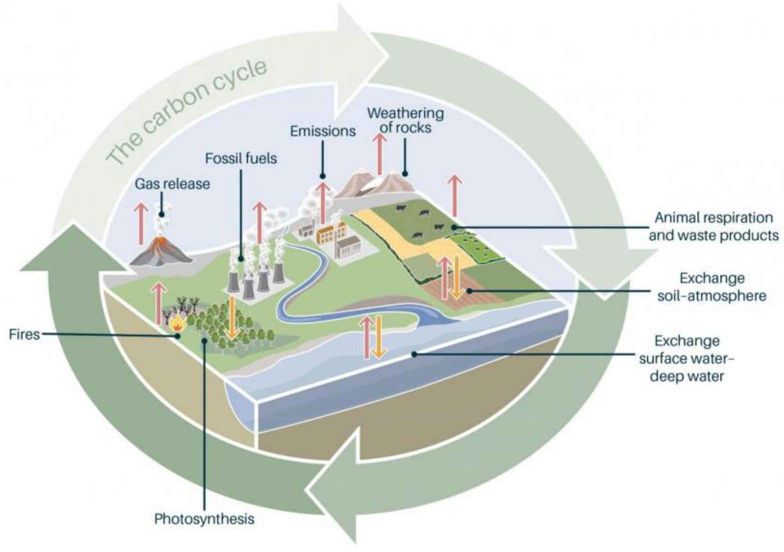

Bgs.ac.uk

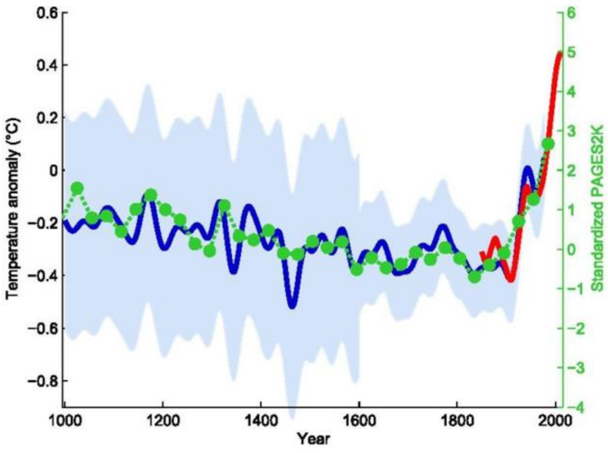

Wikipedia.org

Background

Research seeks to develop a ɣ-CD-MOF synthesis strategy, adapted from the literature such that it is suitable for high school students and safe for execution at home.

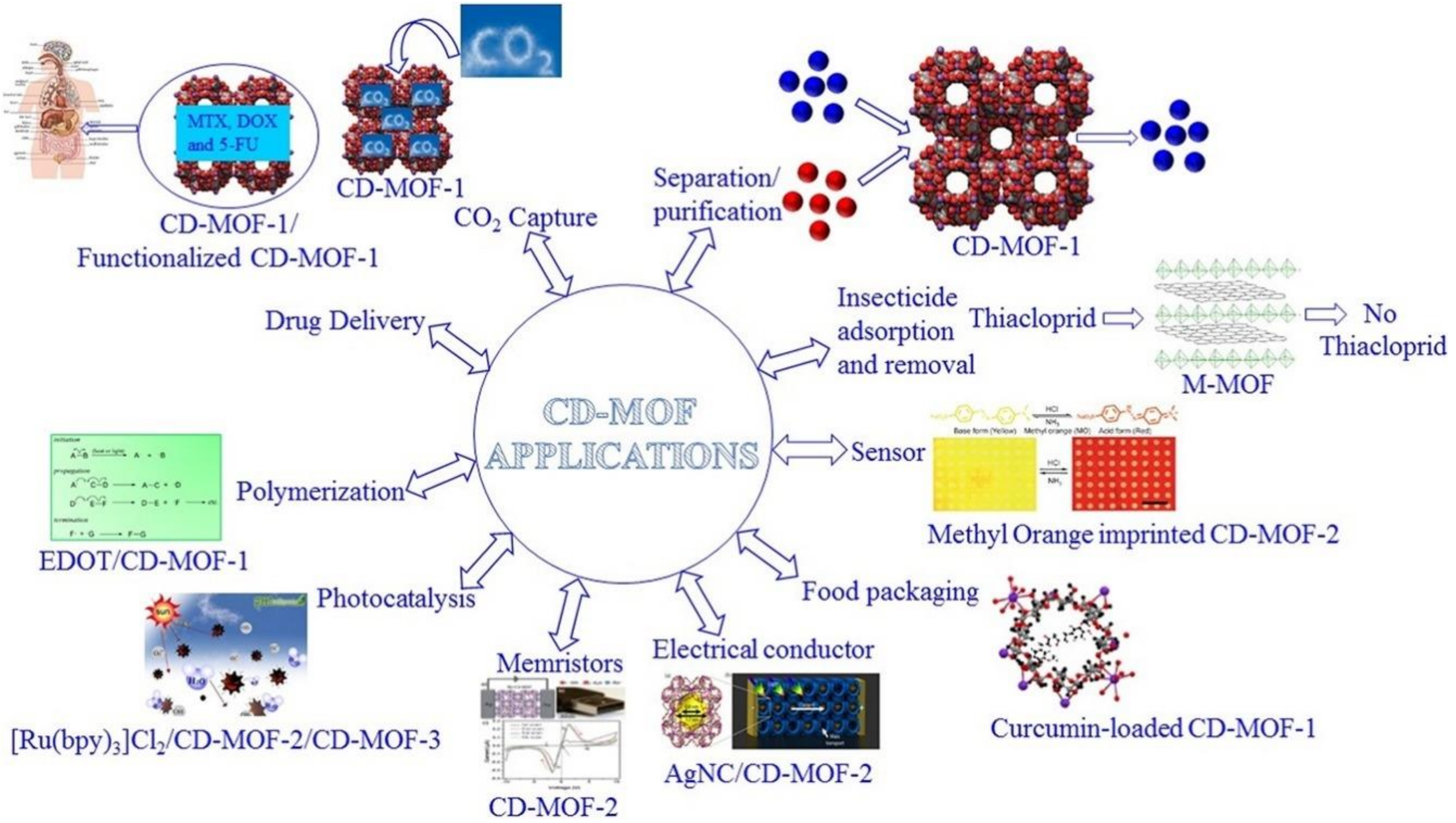

Rajkumar et al. Cyclodextrin-Metal–Organic Framework (CD-MOF): From Synthesis to Applications. *Journal of Industrial and Engineering Chemistry* **2019**, *72*, 50–66.

Student Learning Outcomes

Students will learn the underlying chemistry of framework materials and chemical research techniques.

Environmental Stewardship

- Science as a tool to solve environmental challenges
- Green chemistry
- Real-world applications

Laboratory Techniques

- Vapor diffusion
- Solvent exchange
- Measuring reagents
- Lab safety

Fundamental Chemistry Concepts

- Crystal growth
- Coordination complexes
- Reticular synthesis
- Acid–base chemistry
- Host–guest interaction
- 12 Principles of Green Chemistry

Students at the high-school level will synthesize cutting-edge molecules and test their CO_2 sequestration capabilities, using benign methods and reagents.

Improve equity in the future scientific community.

Green chemistry focuses on designing chemicals and processes to be nonhazardous and sustainable by using the following principles.

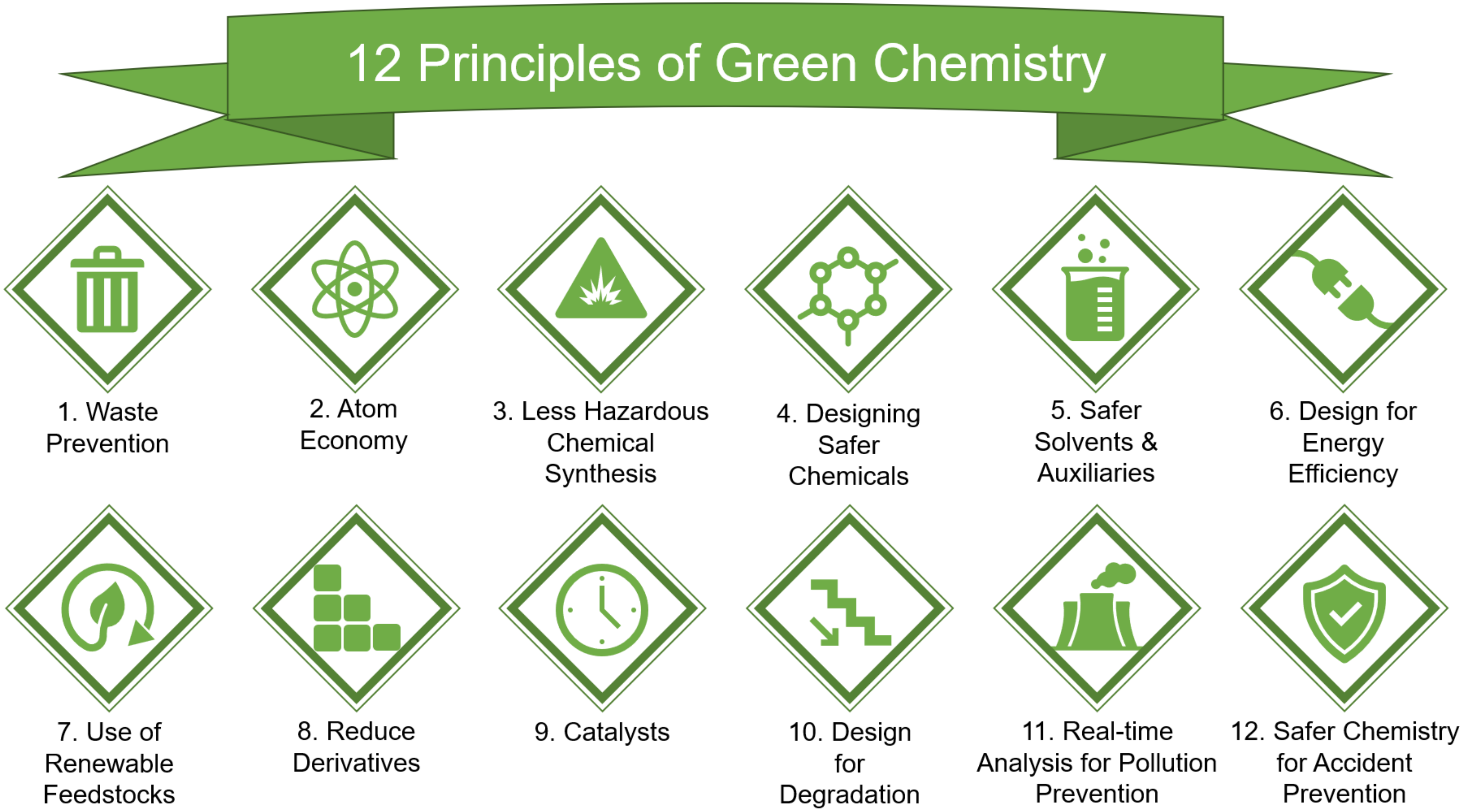

Experimental Design

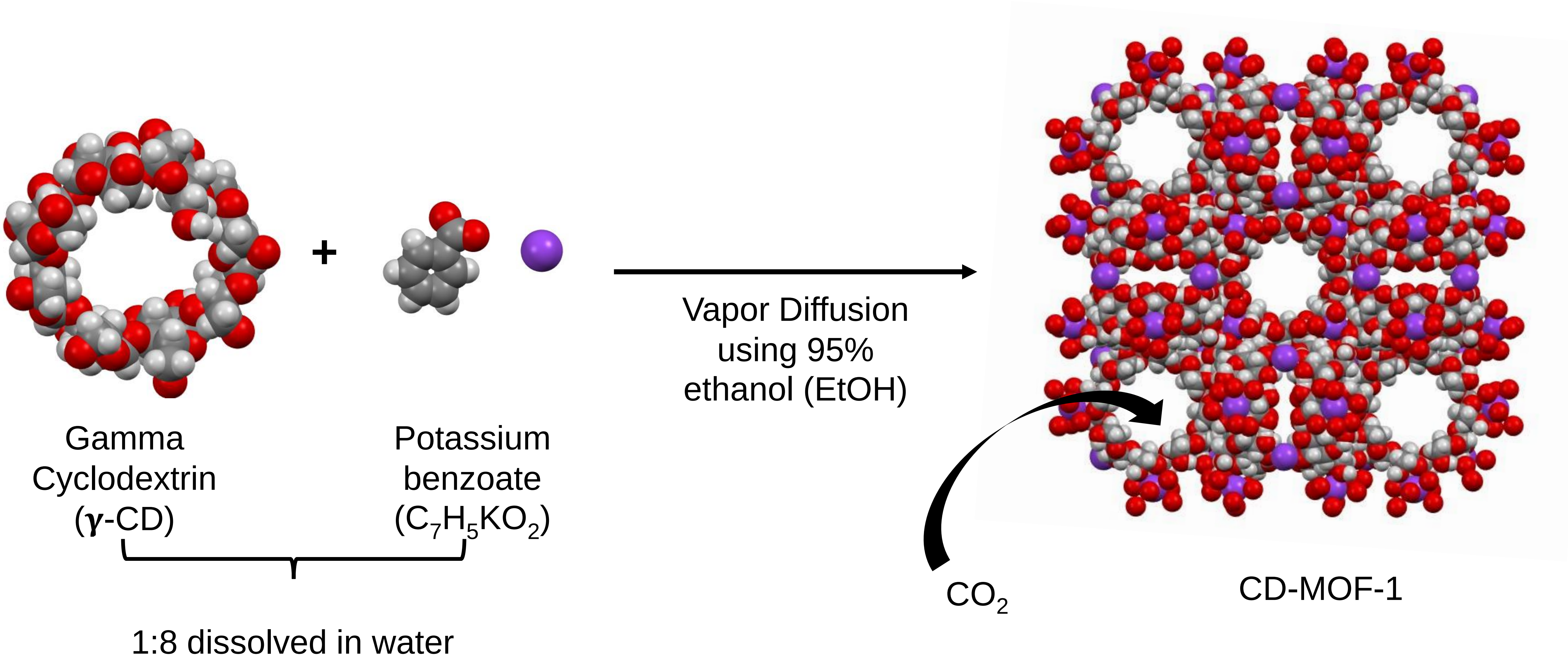

- Results in CD-MOF-1 extended porous framework
- Pores excel at CO_2 uptake

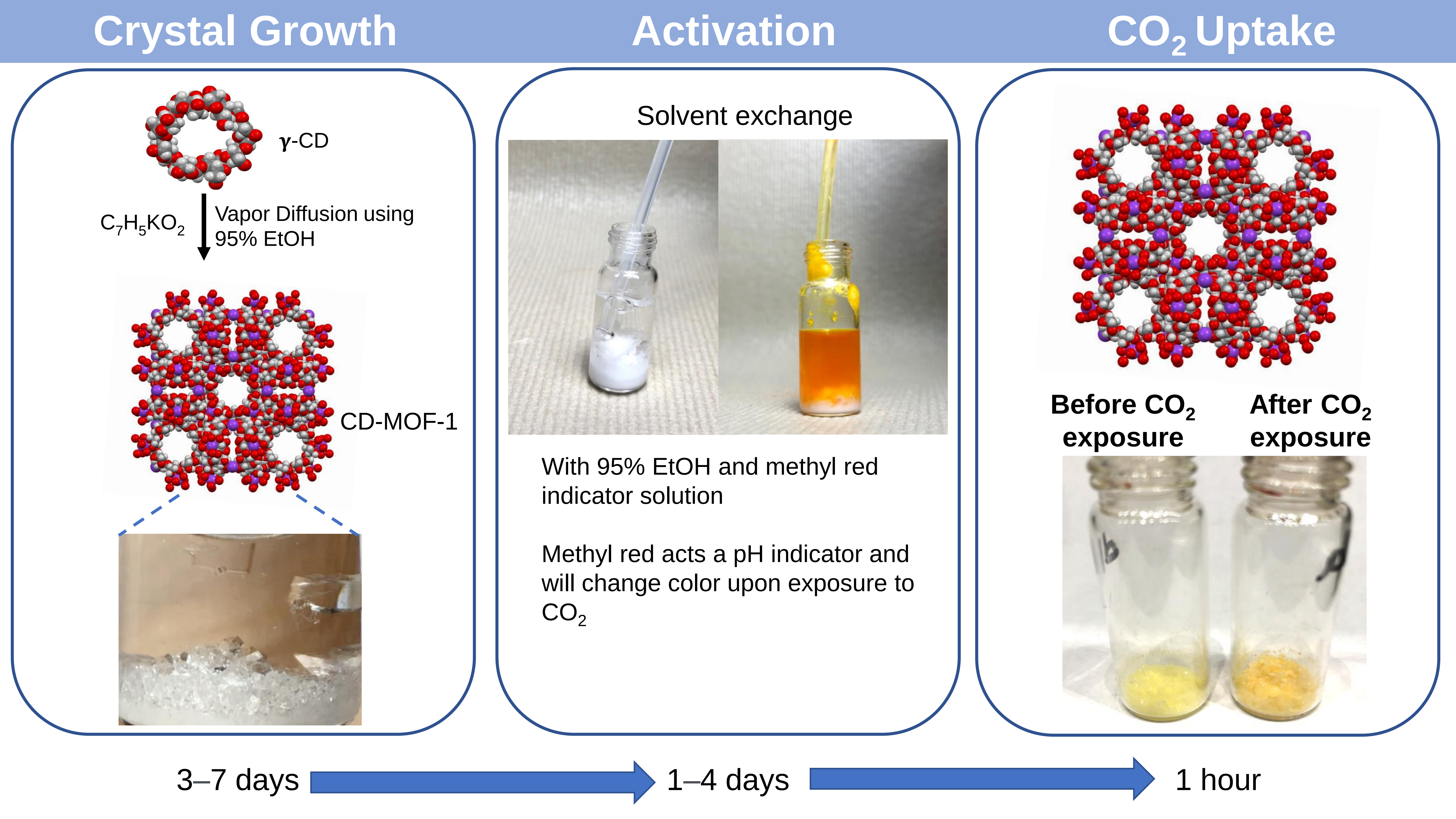

Crystal Growth
Activation
CO_2 Uptake
γ-CD
$C_7H_5KO_2$
Vapor Diffusion using 95% EtOH
CD-MOF-1
Solvent exchange
With 95% EtOH and methyl red indicator solution
Methyl red acts a pH indicator and will change color upon exposure to CO_2
Before CO_2 exposure
After CO_2 exposure
3–7 days
1–4 days
1 hour

Procedure for CD-MOF-1 Crystal Growth

Vapor Diffusion Set Up

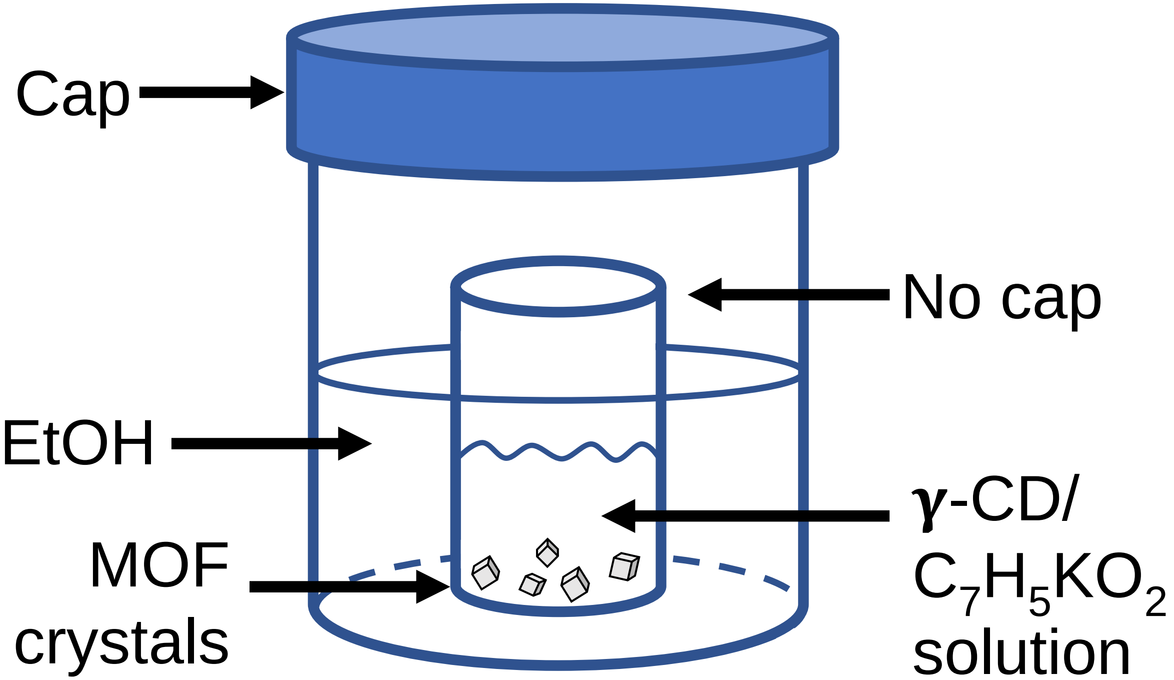

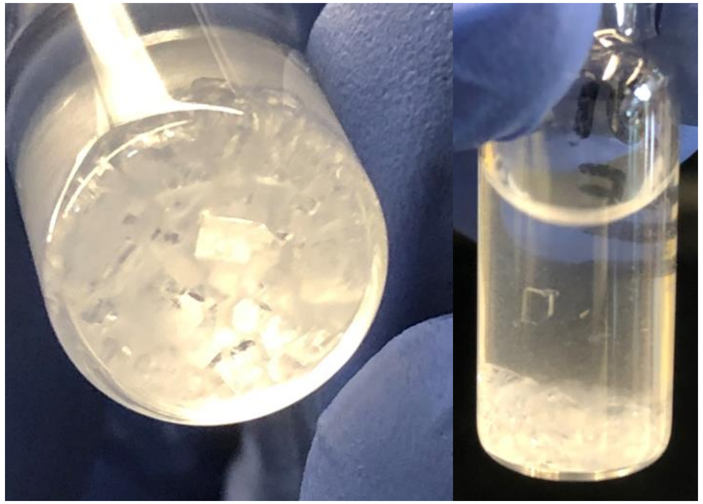

Procedure

- Add gamma cyclodextrin (**γ**-CD) and potassium benzoate ($C_7H_5KO_2$) (1:8 ratio) into DI H_2O
- Filter 1 mL of **γ**-CD/$C_7H_5KO_2$ solution into small vial
- Add 4 mL of 95% EtOH into larger vial
- Add small vial with precursor solution into larger vial and tightly cap outer vial
- Leave for 3–7 days to allow crystal growth

Results

- Clear, cubic MOF crystals (1−3 mm in length)
    - Some white powder (**γ**-CD) precipitates out of solution but can be removed in activation steps

Procedure for CD-MOF-1 Activation and Indicator Incorporation

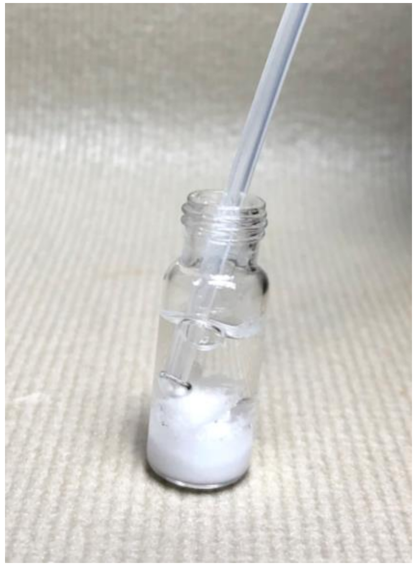

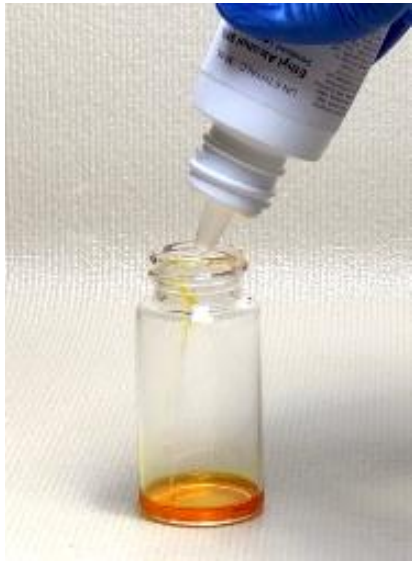

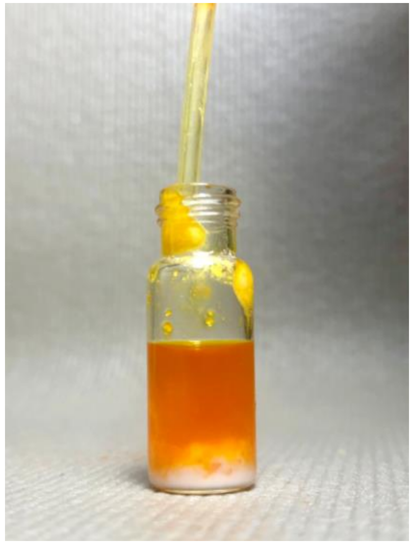

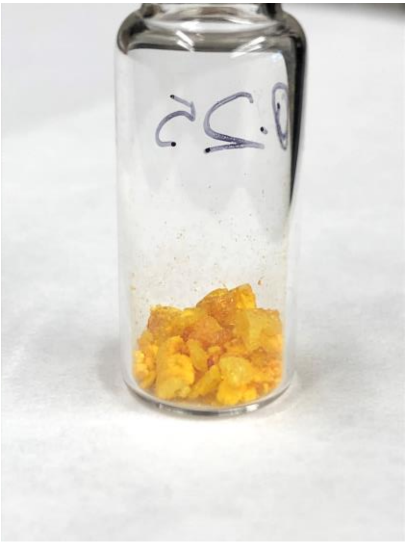

Activation:

- Solvent exchange removes aqueous solvent from MOF pores
- Enables CO_2 uptake in next procedural step

Procedure

- Pipette out solvent surrounding MOF crystals
- Add 1.32 mM methyl red indicator solution in 95% EtOH and let sit for 1–24 hrs then pipette solvent out (repeat)
- Add EtOH 95% to MOF crystals and let sit for 1–24 hrs then pipette solvent out
- Dry slightly capped for 2 days

Results

- MOF crystals successfully dyed yellow
- Indicator incorporated to serve as pH indicator of CO_2 uptake

Procedure for CO_2 Exposure: Qualitative analysis

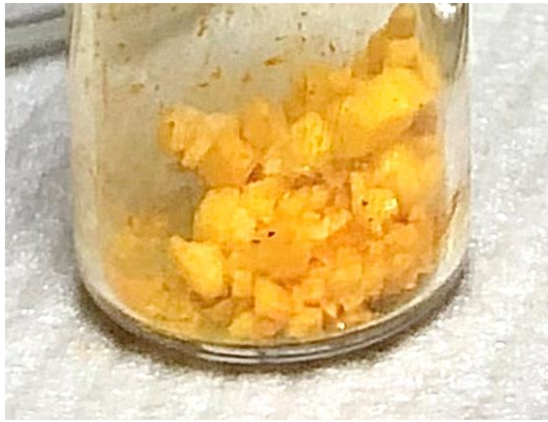

γ-CD MOF before CO_2 exposure

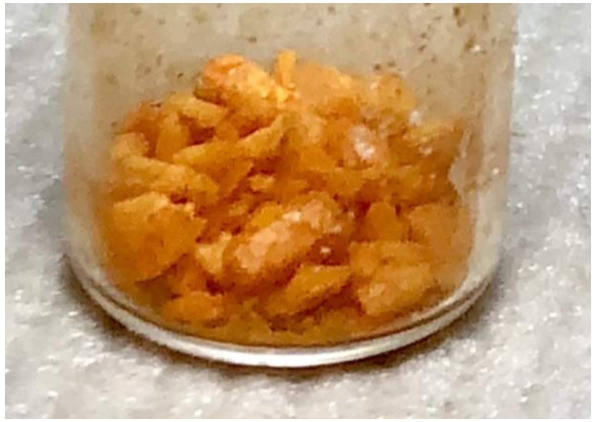

γ-CD MOF after CO_2 exposure

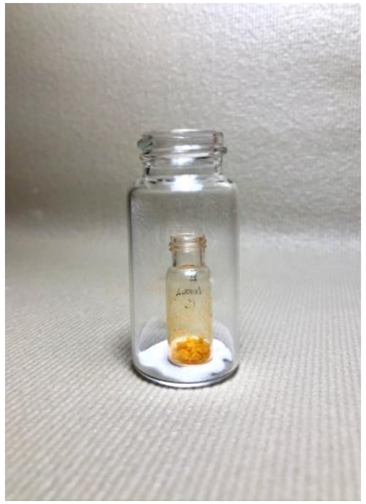

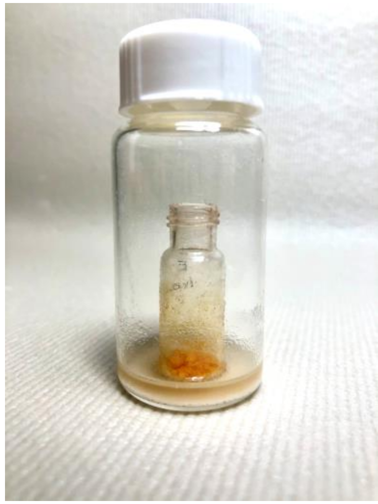

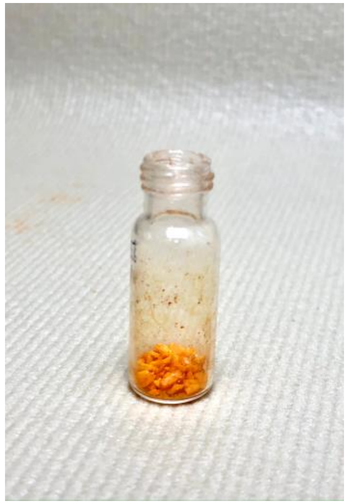

Procedure

- Add baking soda and small vial containing CD-MOF-1 crystals to larger vial
- Pipette vinegar into outer vial and cap
- Observe color change over the next 5–10 minutes

Results

- Qualitative color change of methyl red-activated CD-MOF-1 crystals
    - Yellow to orange indicates CO_2 uptake

- 2 successful experiments using baking soda and vinegar reaction
    - 20 mL of generated CO_2

Overarching Goals

Engaging home-based scientific chemistry with real-world applications

Exposure to environmental stewardship

Hands on experience with laboratory techniques

Introduce fundamental principles of green chemistry and materials chemistry

- Crystal growth
- Coordination complexes
- Reticular synthesis
- Acid–base chemistry
- Host–guest interaction
- 12 Principles of Green Chemistry
